# Anti-distortion bioinspired camera with an inhomogeneous photo-pixel array

**DOI:** 10.1038/s41467-024-50271-7

**Published:** 2024-07-17

**Authors:** Changsoon Choi, Henry Hinton, Hyojin Seung, Sehui Chang, Ji Su Kim, Woosang You, Min Sung Kim, Jung Pyo Hong, Jung Ah Lim, Do Kyung Hwang, Gil Ju Lee, Houk Jang, Young Min Song, Dae-Hyeong Kim, Donhee Ham

**Affiliations:** 1 John A. Paulson School of Engineering and Applied Sciences, Harvard University, Cambridge, MA 02138 USA; 2 Center for Opto-Electronic Materials and Devices, Post-silicon Semiconductor Institute, Korea Institute of Science and Technology (KIST), Seoul, 02792 Republic of Korea; 3 Center for Nanoparticle Research, Institute for Basic Science (IBS), Seoul, 08826 Republic of Korea; 4 School of Chemical and Biological Engineering, Institute of Chemical Processes, Seoul National University, Seoul, 08826 Republic of Korea; 5 School of Electrical Engineering and Computer Science, Gwangju Institute of Science and Technology, Gwangju, 61005 Republic of Korea; 6 KU-KIST Graduate School of Converging Science and Technology, Korea University, Seoul, 02792 Republic of Korea; 7 Division of Nanoscience and Technology, KIST School, University of Science and Technology (UST), Seoul, 02792 Republic of Korea; 8 Department of Electronics Engineering, Pusan National University, Busan, 46241 Republic of Korea; 9 Center for Functional Nanomaterials, Brookhaven National Laboratory, Upton, NY 11973 USA

**Keywords:** Sensors and biosensors, Optoelectronic devices and components

## Abstract

The bioinspired camera, comprising a single lens and a curved image sensor—a photodiode array on a curved surface—, was born of flexible electronics. Its economical build lends itself well to space-constrained machine vision applications. The curved sensor, much akin to the retina, helps image focusing, but the curvature also creates a problem of image distortion, which can undermine machine vision tasks such as object recognition. Here we report an anti-distortion single-lens camera, where 4096 silicon photodiodes arrayed on a curved surface in a nonuniform pattern assimilated to the distorting optics are the key to anti-distortion engineering. That is, the photo-pixel distribution pattern itself is warped in the same manner as images are warped, which correctively reverses distortion. Acquired images feature no appreciable distortion across a 120° horizontal view, as confirmed by their neural-network recognition accuracies. This distortion correction via photo-pixel array reconfiguration is a form of in-sensor computing.

## Abstract (web summary)

Curvature of image sensors that match the focal plane of the lens facilitate focussing but can cause image distortion. Here, an anti-distortion single-lens camera was developed using a curved image sensor with a photo-pixel distribution pattern warped like image warping to correctively reverse distortion.

## Introduction

Many animal eyes, including our own, have evolved a remarkably economical optical system, which comprises a single lens and a retina that is curved to fit the curved focal plane of the lens^1–4^. A wealth of efforts has been dedicated to engineering this structural simplicity into a camera, by combining a single lens and a curved image sensor, *i.e*., a photodiode array on a curved surface realized through flexible electronics fabrication techniques^5–8^. The resulting savings in size and cost of such bioinspired cameras is attractive for space- or weight-constrained machine vision applications, such as in miniature mobile robots, small self-flying vehicles, and tiny distributed sensors connected through communication networks^9,10^.

While the curved surface of the image sensor that matches the focal plane of the lens in the bioinspired single-lens camera helps focus images^11,12^, this very curvature creates its own problem: image distortion^13^. The distortion arises because the coordinates of an arbitrary ray arrival point on the curved sensor surface are nonlinearly scaled from the coordinates of the corresponding ray origin point on an object. This image distortion, which is particularly pronounced in wide field-of-view (FoV) imaging^14^, can undermine machine vision tasks, such as object recognition and subsequent self-navigation^15,16^. One remedy is to ‘de-warp’ the warped image in back-end digital processors before performing object recognition, but this approach comes at the expense of increased computational intensity, power consumption, and data processing latency^17^.

**Fig. 1:**
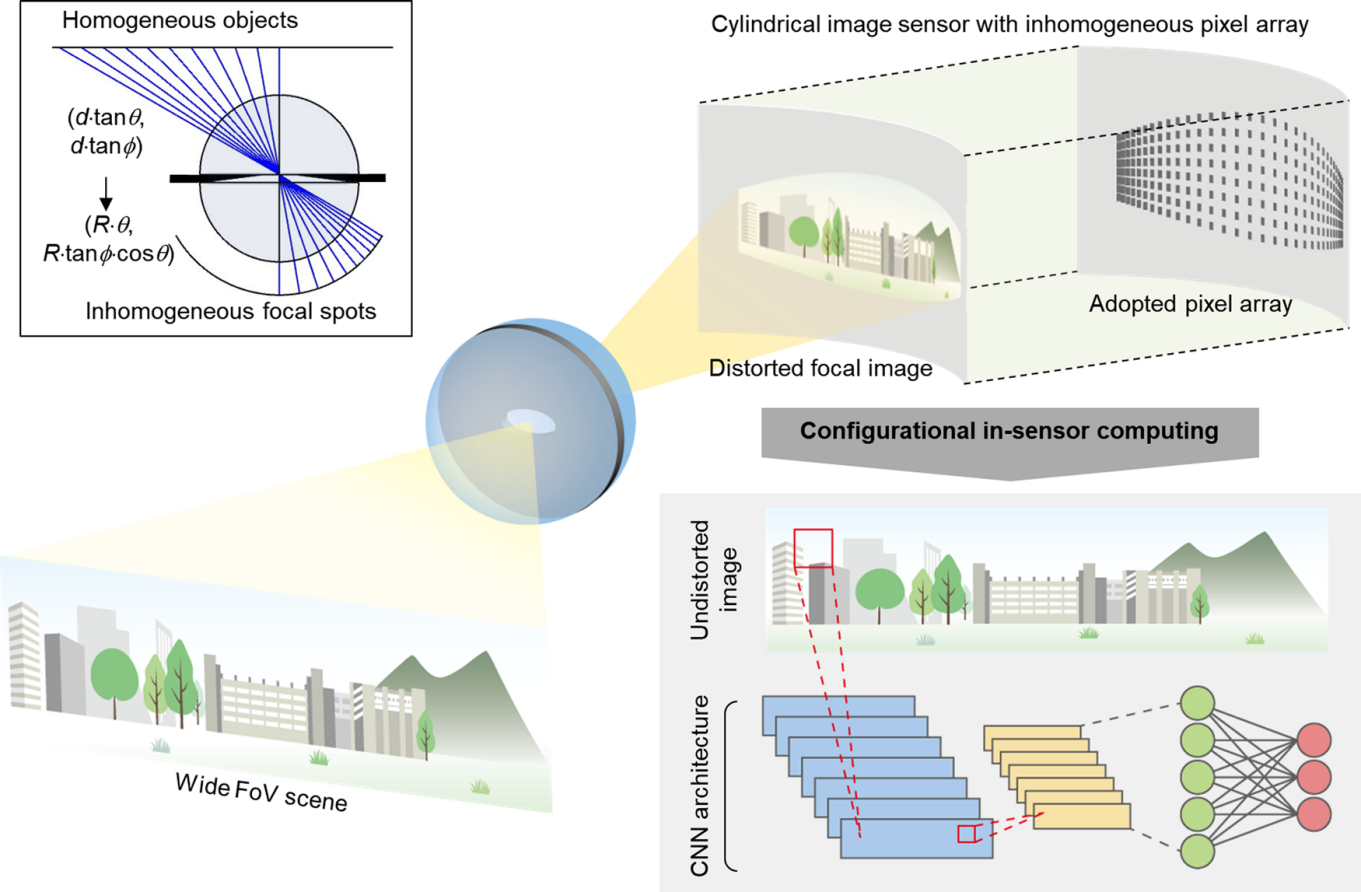
Conceptual illustration of anti-distortion bioinspired camera. In a single-lens bioinspired camera, the curved sensor surface distorts images by causing nonlinear coordinate scaling between ray origin and destination points (inset). Distribution of silicon photodiodes on the curved sensor surface in an inhomogeneous pattern assimilated to the nonlinear coordinate scaling can offset the distortion. That is, by warping the photo-pixel distribution pattern in the same way as the image is warped on the curved surface, image distortion can be preemptively corrected. This is a configurational in-sensor computing, since the pixel distribution itself performs the corrective reversal of the distorting optics. The lack of image distortion facilitates machine vision tasks, such as accurate object recognition by neural networks following the image acquisition.

Here we report an anti-distortion bioinspired camera comprising a single spherical lens and a curved image sensor. The key to the anti-distortion engineering is to distribute silicon photodiodes on the curved sensor surface not uniformly, as in the standard practice, but in an inhomogeneous pattern that is assimilated to the nonlinear coordinate scaling (Fig. 1). In other words, the photo-pixel distribution pattern is warped in the same fashion as images are warped on the curved sensor surface. Then, the back-end digital signal processor, which treats the pixels as if they were uniformly distributed, perceives undistorted images at no extra computational cost. This approach, wherein the preemptive correction of image distortion is performed within the image sensor itself via the non-uniform configuration of the photo-pixel array, may be considered a form of in-sensor computing, since the pixel distribution itself performs the corrective reversal of the distorting optics (Fig. 1). While in-sensor computing so far has been largely focused on harnessing the physics of optoelectronic conversions^18,19^ and photocurrent accumulations^20–23^ for arithmetic operations^24–27^, this work adds another strategy to in-sensor computing, where structural reconfiguration of an image sensor—*i.e*., inhomogeneous pixel arraying—can counterbalance the distorted optics.

**Fig. 2:**
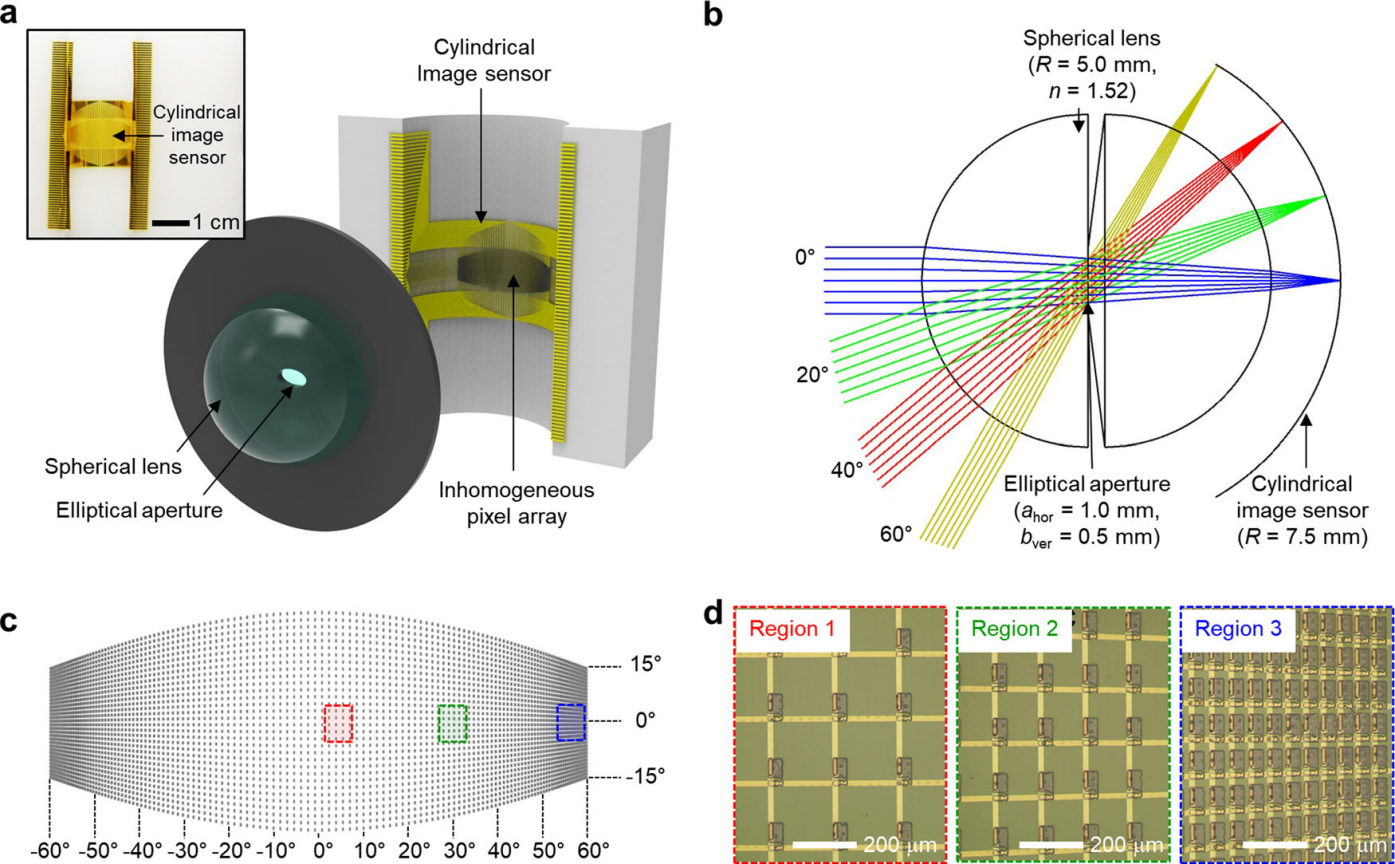
Inhomogeneous pixel array on a cylindrical surface. **a** Schematic illustration of the anti-distortion bioinspired camera, consisting of a spherical lens, an elliptical aperture, and a cylindrical image sensor with an inhomogeneous pixel array. Inset shows a photo of the fabricated cylindrical image sensor. **b** Top view schematic of the camera. **c** Schematic illustration of the inhomogeneous pixel array in a cropped oval boundary. **d** Optical micrographs of inhomogeneously distributed pixels in three different regions of the cylindrical image sensor.

To construct the image sensor, we follow a standard flexible electronics fabrication recipe^28,29^, in which an inhomogeneous silicon nanomembrane photodiode array and its interconnects are fabricated on a planar wafer, and subsequently transferred onto a curved surface^5,12^. The concept of inhomogeneous pixel arraying for anti-distortion engineering is general and can be applied to any curved sensor surface. In this work, we choose a cylindrical surface for the image sensor to facilitate the proof of concept; as the cylindrical surface causes no wrinkle formation during transfer, the inhomogeneous pixel arraying can be tested at high pixel density. In contrast, spherical surfaces suffer from wrinkle formation^30^, unless provisions for large inter-pixel spaces are made at the cost of pixel density^4,31^. On a cylindrical surface, since the vertical range that matches the curved focal plane of the spherical lens is somewhat limited, we set the pixel array area to what corresponds to a wide horizontal FoV (–60° ~ 60°) and a narrower vertical FoV (–15° ~15°). It is within this area that we inhomogeneously distribute 128 (h) × 32 (v) = 4096 silicon photodiodes (Fig. 2c). Our camera so constructed captures MNIST handwritten digit images with no apparent distortion, regardless of their positions. Thanks to this lack of distortion, the convolutional neural network (CNN) based recognition of the images acquired from near the center (0° ~ 30°), middle (30° ~ 45°), and edge (50° ~ 60°) of the horizontal FoV yields similar accuracies of 88%, 93%, and 86%, respectively.

This work forges a connection between in-sensor computing and bioinspired cameras, demonstrating configurational in-sensor computing—*i.e*., preventive correction of image distortion via systematically non-uniformized pixel populations—for a wide FoV bioinspired camera. In the future, the demonstrated strategy may be applied to other curved sensor geometries, most notably, spherical surfaces, to enable anti-distortion wide FoV imaging across not only horizontal, but also vertical directions, by employing emerging flexible electronics fabrication methods, such as kirigami techniques^4,32^, that are less fraught with wrinkle formation.

## Results

### Inhomogeneous pixel arraying for anti-image distortion

Our camera consists of a single spherical lens, an elliptical aperture, and a cylindrical image sensor featuring an inhomogeneous pixel array (Fig. 2a). Fig. 2b shows the geometry of our camera construct viewed from the top with several horizontal incident angles illustrated. The center of the spherical lens (radius ≈ 5.0 mm) lies on the axis of the cylindrical image sensor (radius ≈ 7.5 mm) to the first-order approximation. At the center of the spherical lens, which comprises two hemispherical lenses (BK7; refractive index ~1.52), lies an elliptical aperture (horizontal major axis: 1.0 mm; vertical minor axis: 0.5 mm). See Supplementary Table 1 for structural and material details of the system construct.

A ray originating from a point on an object plane at a horizontal angle of *θ* and a vertical angle of *ϕ,* whose extremities define the horizontal and vertical FoVs, ends up on a point on the cylindrical sensor surface after passing through the aperture at the center of the spherical lens (see Supplementary Fig. 1). On the one hand, the horizontal and vertical coordinates of the ray origin point on the object plane are (*h*_1_, *v*_1_) = (*d*·tan*θ*, *d*·tan*ϕ*), where *d* is the distance of the plane from the spherical center. On the other hand, the horizontal and vertical coordinates of the corresponding ray destination point on the cylindrical sensor surface are (*h*_2_, *v*_2_) = (*R*·*θ*, *R*·tan*ϕ*·cos*θ*), where *R* is the radius of the cylinder (as noted earlier, the spherical center is on the cylindrical axis to the first order approximation, and the derivation assumes the spherical center on the cylindrical axis). Thus, the horizontal and vertical coordinate ratios, *h*(*θ*) ≡ *h*_2_/*h*_1_ = (*R/d*)·(*θ/* tan*θ*) and *v*(*θ*) ≡ *v*_2_/*v*_1_ = (*R/d*)·(cos*θ*), are not constants, but rather are functions of *θ*, and hence the destination coordinates are nonlinearly scaled from the origin coordinates. Any curved geometry of the sensor would cause nonlinear coordinate scaling; the specific expressions of *h*(*θ*) and *v*(*θ*) above, which depend only on *θ* but not on *ϕ*, arise from the geometric character of the cylindrical surface with zero Gaussian curvature. Importantly, this nonlinear coordinate scaling is the cause of image distortion. For example, a uniformly distributed horizontal line of ray origin points across the object plane will yield corresponding line of destination points on the cylindrical sensor surface, whose distribution is not uniform, but rather more compressed towards the edges of the line in a manner according to *h*(*θ*) (Fig. 1, inset).

To offset this image distortion characterized by *h*(*θ*) and *v*(*θ*), we inhomogeneously distribute 128 (h) × 32 (v) = 4096 pixels—each of which is a *p*-*i*-*n* silicon photodiode—with their horizontal and vertical distribution densities proportional to *h*(*θ*) and *v*(*θ*), respectively (Fig. 2c, d). This inhomogeneous arraying is done within the cropped oval boundary on the cylindrical sensor surface, as shown in Fig. 2c. The sensor surface area within this boundary is the region that receives light from across the rectangular FoV with the maximum |*θ* | of 60° and maximum |*ϕ* | of 15° (in other words, the boundary corresponds to (*h*_2_, *v*_2_) computed with varying *ϕ* for *θ*  = ± 60° and varying *θ* for *ϕ* = ± 15°). As an image arriving on the sensor surface is warped according to *h*(*θ*) and *v*(*θ*), the pixel distribution pattern defined by *h*(*θ*) and *v*(*θ*) is also warped exactly in the same quantitative manner as the image warping. Therefore, the back-end digital processor, which assumes as if the pixels were uniformly distributed at no additional computational expense, perceives an undistorted image. For example, a rectangular scene filling the full FoVs will form a cropped oval image on the cylindrical surface according to *h*(*θ*) and *v*(*θ*), but the pixel array whose pattern is defined also by *h*(*θ*) and *v*(*θ*) produces an undistorted, rectangular image. In sum, the key to achieving the anti-distortion imaging with a single-lens, curved-sensor system without back-end compensation (Supplementary Fig. 2) is the mathematically reconfigured distribution of the pixels, which preemptively corrects the image distorting optics.

Before concluding this section, we note that while ref. ^33^ also reports inhomogeneous distribution of pixels, this previous work is fundamentally different from the present work in both purpose and design. Specifically, the previous work concentrated pixels more densely at the center of the sensor surface to achieve a higher resolution therein, has thus nothing to do with correcting image distortion, and employs no specific mathematical formulation for the inhomogeneous arraying of pixels.

### Fabrication and operation of the cylindrical image sensor

**Fig. 3:**
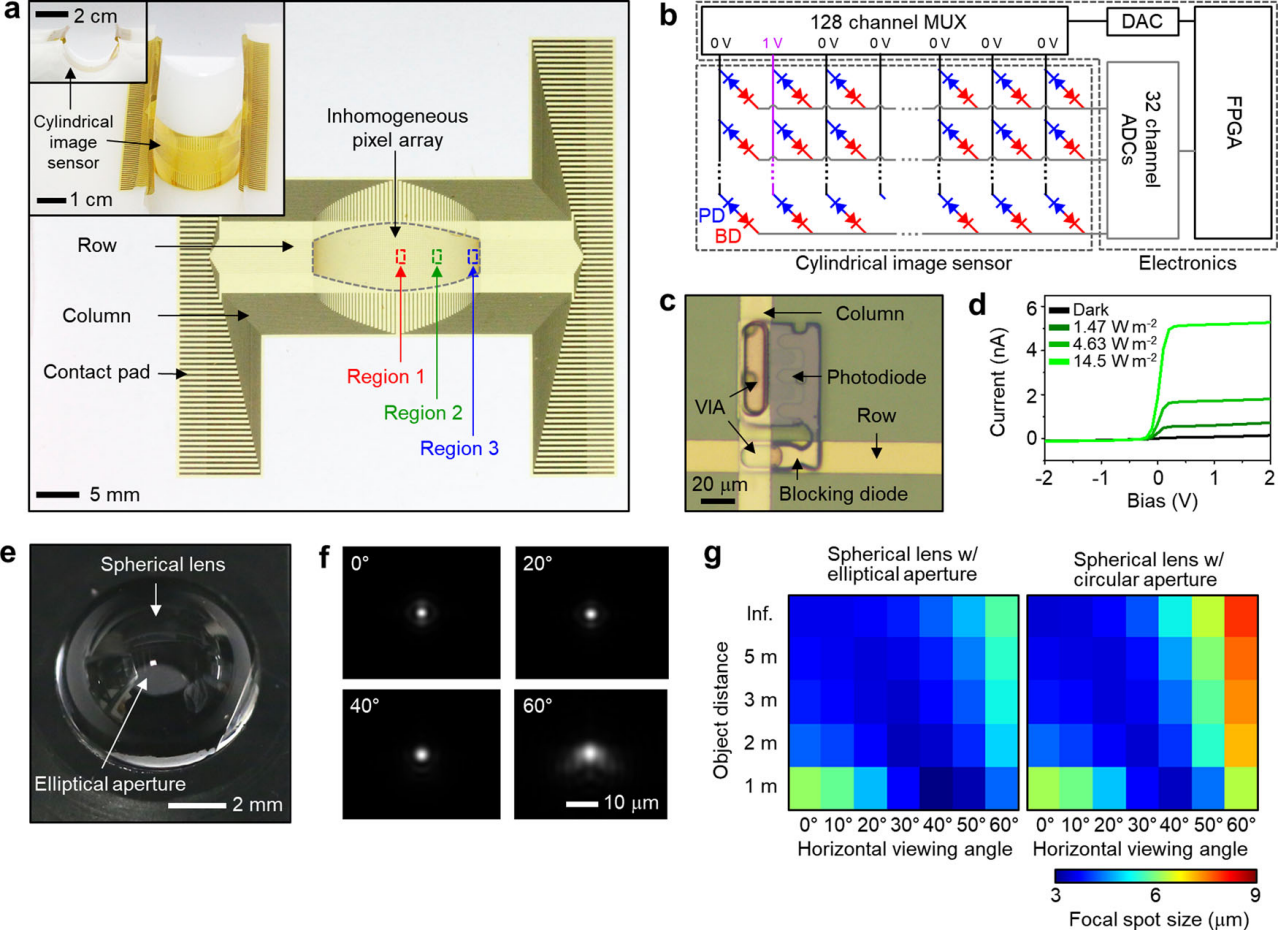
Electronic operation and aperture optics of the camera. **a** Photograph of the non-uniform 128 × 32 silicon photodiode array. Inset: photograph of the cylindrical image sensor. **b** Electronics setup to read out pixel photocurrents for real-time image acquisition. **c** Optical micrograph of a single pixel comprising a photodiode (PD) and a blocking diode (BD) that are back-to-back connected. **d** Measured current vs. bias voltage of a representative pixel, under light illumination with various intensities (light source: 532-nm laser diode). Its responsivity is 0.10 A W^−1^ for an intensity of 1.47 W m^−2^. The results for a wide range of intensities, spanning from 7.14 × 10^−3^ W m^−2^ to 1.52 × 10^5^ W m^−2^, are shown in Supplementary Fig. 6e. **e** Photograph of the spherical lens and the elliptical aperture. **f** Point spreading functions (simulations) of our camera for varying horizontal viewing angles. **g** Simulated focal spot size as functions of object distance and horizonal viewing angle, for the anti-distortion bioinspired camera with the elliptical aperture (1.0 mm major axis and 0.5 mm minor axis) used in our work (left) and with a circular aperture with a diameter of 1.88 mm (right).

We utilize flexible electronics fabrication techniques to realize this image sensor with inhomogeneously distributed pixels on the cylindrical surface (Fig. 3, Supplementary Fig. 3, Methods). We first dope *p* and *n* regions to define 4096 *p-i-n* photodiodes in 128 × 32 positions prescribed by *h*(*θ*) and *v*(*θ*) on a 1.25 μm thick silicon nanomembrane atop a silicon-on-insulator (SOI) wafer, and subsequently detach the nanomembrane and place it on a SiO_2_ wafer coated with a polyimide (PI) film. It is at this stage that we wire the 4096 diodes into a 128 × 32 crossbar array, by etching out non-diode regions from the nanomembrane, and then fabricating alternating layers of PI films and lateral metallic interconnects. The lateral metallic interconnects, provided through two layers, are made from Cr/Au (thickness: ~10/100 nm). The PI films are to insulate the two metallic layers and the photodiode layer from one another, and also to provide a finishing layer to passivate the sensor. The photodiodes and lateral metallic interconnects at different layers are connected by Cr/Au vias (thickness: ~10/100 nm). We then transfer this pliable platform—comprising silicon *p-i-n* photodiodes and metal interconnects with PI passivation at top and bottom—onto a cylindrical PDMS substrate, using the transfer printing technique with a water-soluble tape. This completes the fabrication of the cylindrical image sensor. The transfer of the flexible platform onto the cylindrical surface does not cause wrinkle formation (Fig. 3a, inset), for it is conformable to the cylindrical surface with the zero Gaussian curvature (Supplementary Fig. 4)^33^.

While the foregoing paragraph describing the fabrication process captures the structural essence of the image sensor, its actual structure is in fact more complex, with each pixel of the 128 × 32 crossbar array featuring not only the *p-i-n* photodiode, but also a *p-n* blocking diode, where these two diodes are connected back-to-back by sharing the *p* region in common (Fig. 3b, c). The blocking diode is deliberately placed under a metal interconnect to minimize an exposure to light irradiation (Fig. 3c; Supplementary Fig. 5a). The blocking diode of each pixel acts as a rectifier, suppressing sneak path currents in the crossbar array and thus minimizing interference amongst neighboring pixels (Supplementary Fig. 5b)^34^. In the presence of a light, a positive bias, *V*_*d*_ = 1 V, to a pixel (*i.e*., reverse biasing the photodiode and forward biasing the blocking diode; see Fig. 3b) enables a photocurrent production, whereas a zero bias (*V*_*d*_ = 0 V) to the pixel prevents a photocurrent production by reverse-biasing the blocking diode (Fig. 3d). Supplementary Fig. 6 shows additional photoresponse characterization of a single pixel, such as endurance, response time, and dynamic range.

Figure 3b shows the electronics setup that enables the image sensor to capture real-time images. Concretely, using the 128:1 multiplexer (MUX), we sequentially activate a given column with a positive voltage (*V*_*d*_ = 1 V), while the other columns are disabled by applying a zero-bias (*V*_*d*_ = 0 V). For a given activated column, the photocurrents of its 32 pixels are measured through the 32 rows feeding 32-channel analog-to-digital converters (ADCs). These ADCs directly digitize the photocurrents by measuring integrated charge during each sampling period. With the chosen settings in our highlight experiments, it takes approximately 36 ms for the entire 128 × 32 array to be scanned, and thus the image acquisition rate is ~27 frames per second (fps). The entire image acquisition operation is controlled by a field-programmable gate array (FPGA) (Fig. 3b, Supplementary Fig. 7). See Methods for details of the image acquisition electronics.

Each subfigure of Supplementary Fig. 5c shows photocurrents measured across the pixel array on the cylindrical surface with the custom electronics setup, under spatially uniform light illumination with a given intensity. Not only do these measurements show the working of the electronics setup to read out the photocurrents across the pixel array, but they also reveal quite significant pixel-to-pixel variations, which may be attributed to non-uniform doping of silicon photodiodes, non-uniform contact resistances, and possible fracturing of the silicon nanomembrane during the transfer process. All of these are fabrication issues, which may be mitigated by transitioning to industry-standard fabrication from in-house fabrication.

### Camera construct

We have thus far assumed that all rays pass through the exact center of the spherical lens as if the aperture were infinitesimally small and were open just exactly at that center. For example, the nonlinear coordinate scaling functions, *h*(*θ*) and *v*(*θ*), were obtained under the assumption, thus considering the geometric interplay purely between the cylindrical sensor surface and the spherical lens. However, the aperture size cannot be too small, as such would lead inexorably to a very weak incident light on the sensor surface. On the other hand, if the aperture size grows and allows for more ray pathways around the spherical lens center, focal spot size on the sensor surface would increase, causing blurring in the acquired image^35^. The aperture design thus is a critical consideration in constructing the camera.

The elliptical aperture (Figs. 2a, 3e; horizontal major axis: 1.0 mm; vertical minor axis: 0.5 mm) was designed to optimally navigate the tradeoff associated with the aperture size for our camera with the horizontally wide FoV (–60° ~60°) and the vertically limited FoV (–15° ~15°). The larger width (2.0 mm) of the aperture is to guarantee that sufficient light reaches the deep horizontal ends on the sensor surface (Fig. 3f)^35^ at the expense of increasing focal spot width. The aperture height does not have to be as large, given the limited vertical FoV, and is so set at 1.0 mm; in fact, the smaller aperture height keeps focal spot height reasonably small. As we compare, in simulation, the focal spot size (the average of focal spot height and width) for this elliptical aperture and that for a 1.88 mm diameter circular aperture, the former remains the same as or smaller than the latter at all horizontal viewing angles (Fig. 3g; Supplementary Fig. 8), justifying the use of elliptical geometry over circular geometry for the aperture. Note here that the 1.88 mm diameter for the circular aperture is chosen because for a given source light, the destination light intensity distribution on the cylindrical sensor surface across the entire horizontal FoV remains the same between the elliptical aperture and the circular aperture with that diameter.

### Anti-distortion wide FoV imaging

**Fig. 4:**
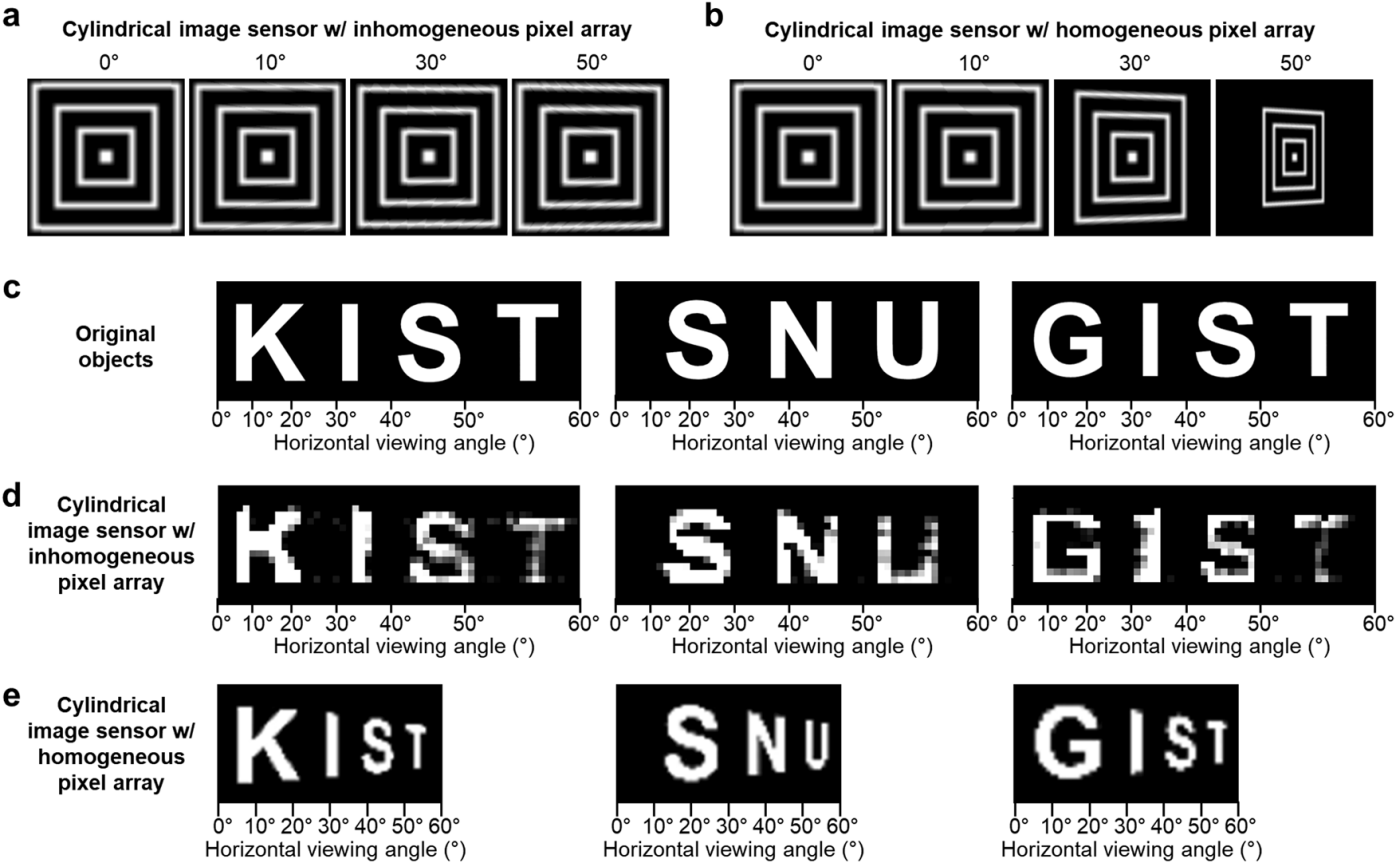
Anti-distortion imaging. **a**, **b** Simulated images of grid objects acquired by the cylindrical image sensor with the inhomogeneous (part **a**) and a homogeneous (part **b**) pixel array for various horizontal viewing angles. **c**–**e** Illustration of three objects, with each spread across the horizontal FoV (0° ~ 60°) (part **c**), their images experimentally acquired by our camera with the cylindrical image sensor with the inhomogeneous pixel array (part **d**), and simulated object images that would be acquired by the cylindrical image sensor with a homogeneous pixel array (part **e**).

Before imaging experiments, we perform three-dimensional (3D) ray tracing simulations to compare the images acquired by the inhomogeneous pixel array and those by the homogeneous pixel array, with both arrays being on the cylindrical surface. Fig. 4a, b juxtapose these simulated sensor-acquired images for objects located on the same plane but at different horizontal viewing angles (*θ* = 0°, 10°, 30°, and 50°). As can be clearly seen, the images acquired by the homogeneous pixel array show warping that increases with *θ* (Fig. 4b), whereas the images acquired by the inhomogeneous pixel array hardly exhibit such warping even at the largest *θ* of 50° (Fig. 4a).

Experimental imaging utilizing our single-lens camera with the inhomogeneous silicon *p-i-n* photodiode array on the cylindrical image sensor is first performed on three ‘objects’ on a plane shown in Fig. 4c; each object is a set of letters spread across the right half of the horizontal FoV (0° to 60°), and the plane is located at a 7 cm distance from the center of spherical lens. See Methods and Supplementary Fig. 7b for the experimental setup. Figure 4d shows the imaging results. As can be seen, the image experimentally acquired for each of the three objects seldom shows distortion even for *θ* approaching 60°. This contrasts the 3D ray tracing simulation results for a camera with the same lens, but with a homogenous pixel array on the same cylindrical surface, where each imaging result is increasingly warped with growing *θ* (Fig. 4e). In Fig. 4d, the intensity reduction at larger viewing angles is vignetting^33^. This intensity attenuation, which is proportional to the first power of the cosine function of the incident angle in our camera with its symmetrical optics, is less severe than the cosine-fourth-power law intensity reduction of traditional cameras.

**Fig. 5:**
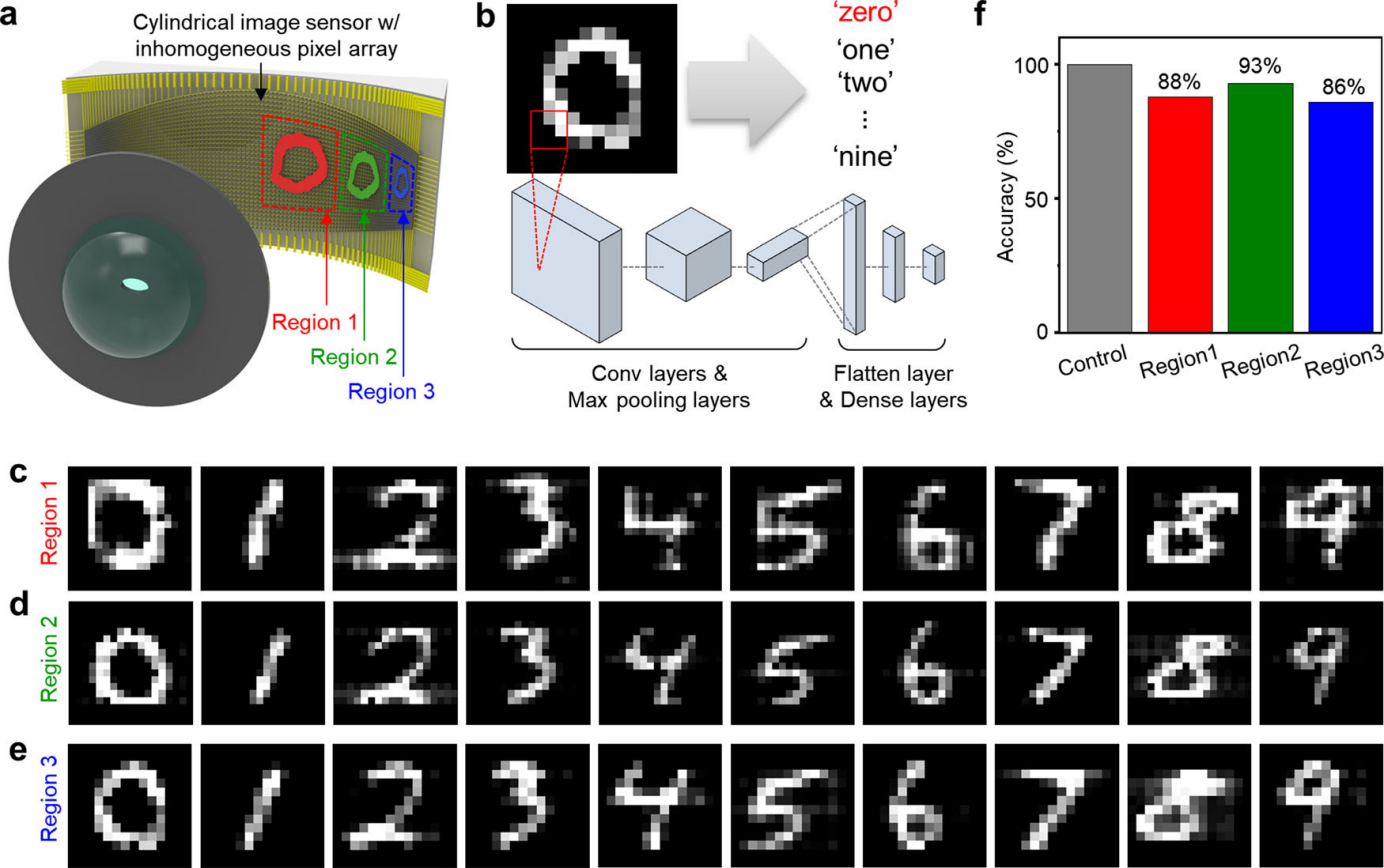
Neural network recognition of camera-acquired MNIST handwritten images. **a** Schematic illustration of the focal image formation of a light pattern (*i.e*., zero) on the cylindrical image sensor. Light patterns incident from three different horizontal viewing angle ranges, 0° ~ 30°, 30° ~ 45°, and 50° ~ 60° hit the three different sensor regions shown as Regions 1, 2, and 3. **b** Illustration of CNN inferencing on an acquired image (for the details of the CNN, see Methods). **c**–**e** Examples of MNIST handwritten digit images experimentally captured in Regions 1, 2, and 3 on the cylindrical image sensor. **f** CNN recognition accuracies for 100 images each acquired in Regions 1, 2, and 3.

Images of objects acquired by the anti-distortion engineered inhomogeneous pixel array must then be able to be recognized by a neural network with consistent accuracies, regardless of where the objects lie in the FoV. To experimentally demonstrate this, as shown in Fig. 5, we treat 100 MNIST handwritten digits as individual objects, and we use our camera to image each of these 100 objects placed at 3 different horizontal positions, 0° ~ 30°, 30° ~ 45°, and 50° ~ 60°, on a plane 4.5 cm away from the center of the spherical lens (the corresponding image formation regions on the cylindrical imager surface are marked as Regions 1, 2, and 3 in Fig. 5a). Subsequently, we run a CNN inferencing algorithm to recognize each acquired image as a digit (Fig. 5b; Methods).

The entire set of 300 acquired images are shown in Supplementary Figs. 9–11. Select examples are in Fig. 5c–e, where images of ten handwritten digits, from zero to nine, captured in the three different regions are displayed. As seen, the images captured in Region 3 hardly show distortion, resembling those captured in Regions 1 and 2. Due to no appreciable distortion, the CNN inferencing recognizes 100 images each in Regions 1, 2, and 3 with similar accuracies of 88%, 93%, and 86% (Fig. 5f). While these accuracies are altogether rather limited due to image degradations caused by pixel-to-pixel variations (including pixel failures) in optoelectronic conversion performance, to appreciate the benefit of anti-distortion imaging, one should rather examine the relative differences—or lack thereof—amongst the recognition accuracies for the three different horizontal regions. Images acquired at Regions 1 and 3 are recognized with similar average accuracies (88% and 86%), and images acquired at Region 2 are recognized with even a higher average accuracy (93%) than images acquired at Region 1 (88%). The comparable recognition accuracies amongst the three regions with no appreciable inferiority at larger viewing angles as compared to small viewing angles attest to anti-distortion imaging and its usefulness from the point of view of machine vision. We also note that the relatively higher recognition accuracy (93%) for images acquired at Region 2 is due probably to the fact that Region 2 has relatively less pixel-to-pixel variations, as seen in Supplementary Fig. 5c.

To further substantiate our findings, we provide a couple more Supplementary Figures. In Supplementary Fig. 12, we perform simulations of the capture of more complex image (fashion image) at different horizontal viewing angles for the cylindrical image sensor with the inhomogeneous and homogeneous pixel arrays, with the former demonstrating no appreciable image distortion, thus lending itself to better CNN recognition accuracies. Supplementary Fig. 13 shows the experimental capture of yet another image (goat image) by our camera with the inhomogeneous pixel array to show again its capability for the anti-image distortion. Finally, see Supplementary Table 2 where the specifications of our camera are compared with those of other bioinspired cameras.

## Discussion

The curved retina of an animal eye is an alluring natural solution to acquire well-focused images with a single lens. A wealth of flexible electronics designs are being employed for developing a curved image sensor inspired by the retina, and a camera that combines the curved sensor with a single lens. On the one hand, the simplified hardware scheme afforded by this approach may benefit space or weight constrained machine vision applications, such as in miniature robotics and small autonomous mobility. On the other hand, as one seeks to widen the FoV of such cameras, the problem of image distortion looms large, one that is inherent to the optics associated with the curved geometry of the sensor surface.

In this work, by integrating a spherical lens and a cylindrical image sensor with a non-uniformly distributed silicon photodiode array, we have developed an anti-distortion bioinspired camera capable of capturing undistorted panoramic images across a wide FoV. Vital to the anti-distortion engineering is the non-uniform photodiode distribution in a pattern that follows the optical distortion characteristics of the curved geometry of the sensor. This arrangement of the pixels to enact the anti-distortion compensation function within the image sensor itself may be viewed as a form of in-sensor computing. It is in this sense that this work forges a connection between bioinspired cameras and in-sensor computing.

The anti-distortion imaging based on the array reconfiguration is generally applicable to any curved geometry. We chose the cylindrical sensor surface as a first demonstration vehicle, for the silicon nanomembrane photodiode array on a flexible substrate can readily conform to the cylindrical surface without wrinkle formation. However, this choice constrains the panoramic imaging to the horizontal direction. Going forward, emerging wrinkle-free fabrication methods, such as the kirigami technique, may help expand the distortion correction strategy to a spherical sensor surface, which would enable anti-distortion panoramic imaging in both the horizontal and vertical directions.

## Methods

### Fabrication of cylindrical image sensor

Here we detail the fabrication of the cylindrical image sensor, in connection with Supplementary Fig. 3. A 1.25 μm thick silicon nanomembrane on an SOI wafer (SOITEC) is first spin-coated with an *n*-type spin-on-dopant (SOD) solution (P509, Filmtronics) and annealed at 975 °C for 12 min to create *n*-doped regions. Then, the silicon nanomembrane is spin-coated with a *p*-type SOD solution (B153, Filmtronics) and annealed at 975 °C for 30 min to create *p*-doped regions. Note that before each spin coating, a 300 nm SiO_2_ layer is deposited on the silicon nanomembrane using plasma-enhanced chemical vapor deposition (PECVD) and is subsequently patterned to define spin-coating regions, and after each spin coating and annealing, the SiO_2_ layer and residual SOD solution are removed. The doping profiles are analyzed using dynamic secondary ion mass spectroscopy (IMS 7f-Auto, CAMECA; Supplementary Fig. 3c). The doped silicon nanomembrane is then separated from the SOI wafer and is transferred to a SiO_2_ wafer covered with a PI film, and individual pixels are patterned using photolithography and reactive ion etching. An additional PI film is then spin-coated, cured, and patterned to form holes, which are subsequently filled with Cr/Au that is deposited with thermal evaporation to form vertical interconnects. The resulting Cr/Au layer is ~ 10/100 nm thick, which is patterned using photolithography and wet etching also to define lateral interconnects. These steps were repeated to form two layers of lateral Cr/Au interconnects. An additional PI film is then added for encapsulation. The resulting silicon photodiode array is then transferred onto the cylindrical PDMS substrate with a radius of curvature of 7.5 mm using a wafer-soluble tape (3 M Corp.). This completes the fabrication of the cylindrical image sensor.

### Image acquisition

The image acquisition electronics are designed for real-time measurement of photocurrents from the 128 × 32 photo-pixel array. This electronic system comprises two 16-channel current-input ADCs (DDC316, Texas Instruments) that measure the photocurrents of 32 pixels in each activated column, a digital-to-analog converter (DAC; DAC80501, Texas Instruments) that generates a positive voltage (1 V), and eight 16:2 MUXes (MAX14661, Analog Devices) that supply either the positive voltage (*V*_*d*_ = 1 V) or a zero voltage (*V*_*d*_ = 0 V) to each column for its activation or deactivation (Fig. 3b). An FPGA (XEM7310, Opal Kelly) controls these electronics through a serial peripheral interface (SPI), and sends captured data to the computer via USB. For imaging, a diffusive light source (COB-220113, Sumbulbs Environment Technology Ltd.) is used to provide a uniform white light illumination, and a shadow mask is mounted on the light source to generate an object image. The 128 × 32 photocurrents acquired by the cylindrical image sensor, which is connected to the electronics via an anisotropic conductive film, are rendered on a 128 × 32 image plot using a custom PyQt-based user interface.

### Ray-tracing simulation

The 3D ray tracing simulation using OpticStudio 20.3, Radiant ZEMAX LLC, finds the destination coordinate (*h*_*2*_, *v*_*2*_) on the cylindrical sensor surface in response to an incident ray originating from any arbitrary source coordinate (*h*_*1*_, *v*_*1*_) on an object plane. From this simulation, a lookup table connecting any general (*h*_*1*_, *v*_*1*_) to its corresponding (*h*_*2*_, *v*_*2*_) is obtained. The simulated image acquired by the cylindrical image sensor with the homogeneous pixel array, such as Fig. 4b and Supplementary Fig. 12b, is obtained by specifying the set of coordinates (*h*_*2*_, *v*_*2*_) for a source set of coordinates (*h*_*1*_, *v*_*1*_) using the lookup table. On the other hand, the simulated image acquired by the cylindrical image sensor with the inhomogeneous pixel array, such as Fig. 4a and Supplementary Fig. 12a, is obtained by specifying the set of indices of the pixels located at or nearby the above-found set of coordinates (*h*_*2*_, *v*_*2*_).

### Image recognition

Image recognition is performed using TensorFlow in Python 3.8. The CNN for the handwritten digit recognition (Fig. 5) starts with three convolutional layers. The first two convolutional layers includes 32 and 64 filters, each with 3 × 3 kernel size and ReLU activation function. Following each of these convolutional layers, max pooling is applied with 2 × 2 pool size. The third convolutional layer includes 64 filters, each with 3 × 3 kernel size and ReLU activation function. A flatten layer converts the resulting 3D feature maps into a 1D vector, which then undergoes two final layers sequentially: the first with 64 nodes and ReLU activation function, and the second with 10 nodes and softmax activation function. The CNN, trained over 20 epochs using 50,000 training images from the MNIST database, is used to recognize the handwritten digit images captured by our camera.

The CNN for the fashion image recognition (Supplementary Fig. 12) starts with three sets of convolutional layers. The first set includes two convolutional layers, each having 64 filters with 3 × 3 kernel size and ReLU activation function. The second set includes two convolutional layers, each having 128 filters with 3 × 3 kernel size and ReLU activation function. Following each set, max pooling with 2 × 2 pool size and batch normalization are applied. The third set is one convolution layer having 64 filters with 3 × 3 kernel size and ReLU activation function, and max pooling with 2 × 2 pool size is applied. The resulting 3D feature maps are converted by a flatten layer into a 1D vector, which is fed to two final layers sequentially: the first with 512 nodes and ReLU activation function, and the second with 10 nodes and softmax activation function. The CNN model trained over 20 epochs using 50,000 training images from the fashion-MNIST database is used to recognize the simulated fashion images.

### Supplementary information

Supplementary Information

Peer Review File

## Data Availability

All data are available in the main text or Supplementary Information.

## Code availability

We use Verilog and Python 3.8 to operate the customized electronics setup. We use Python 3.8 for the CNN work. Source codes are available from the corresponding authors upon request.
