# Supplementary material for: Anti-distortion bioinspired camera with an inhomogeneous photo-pixel array
Source: Nat Commun. 2024 Jul 17;15:6021. doi: 10.1038/s41467-024-50271-7 (PMC11255341; doi:10.1038/s41467-024-50271-7)
Supplement: Supplementary file 1 — Supplementary Information [file 41467_2024_50271_MOESM1_ESM.pdf]

Supplementary Information

Anti-distortion bioinspired camera with an inhomogeneous photo-pixel array

Changsoon Choi[1,2†], Henry Hinton[1†], Hyojin Seung[3,4†], Sehui Chang[5†], Ji Su Kim[3,4], Woosang You[3,4], Min Sung Kim[3,4], Jung Pyo Hong[2,6], Jung Ah Lim[2,7], Do Kyung Hwang[2,6], Gil Ju Lee[5,8], Houk Jang[1,9], Young Min Song[5]*, Dae-Hyeong Kim[3,4]*, and Donhee Ham[1]*

[1]*John A. Paulson School of Engineering and Applied Sciences, Harvard University, Cambridge, MA 02138, USA*

[2]*Center for Opto-Electronic Materials and Devices, Post-silicon Semiconductor Institute, Korea Institute of Science and Technology (KIST), Seoul 02792, Republic of Korea.*

[3]*Center for Nanoparticle Research, Institute for Basic Science (IBS), Seoul 08826, Republic of Korea.*

[4]*School of Chemical and Biological Engineering, Institute of Chemical Processes, Seoul National University, Seoul 08826, Republic of Korea.*

[5]*School of Electrical Engineering and Computer Science, Gwangju Institute of Science and Technology, Gwangju 61005, Republic of Korea.*

[6]*KU-KIST Graduate School of Converging Science and Technology, Korea University, Seoul 02792, Republic of Korea.*

[7]*Division of Nanoscience and Technology, KIST School, University of Science and Technology (UST), Seoul 02792, Republic of Korea.*

[8]*Department of Electronics Engineering, Pusan National University, Busan 46241, Republic of Korea.*

[9]*Center for Functional Nanomaterials, Brookhaven National Laboratory, Upton, NY 11973, USA*

† *C. Choi, H. Hinton, H. Seung, and S. Chang contributed equally to this work.*

*To whom correspondences should be addressed.

E-mail: donhee@seas.harvard.edu, dkim98@snu.ac.kr, ymsong@gist.ac.kr

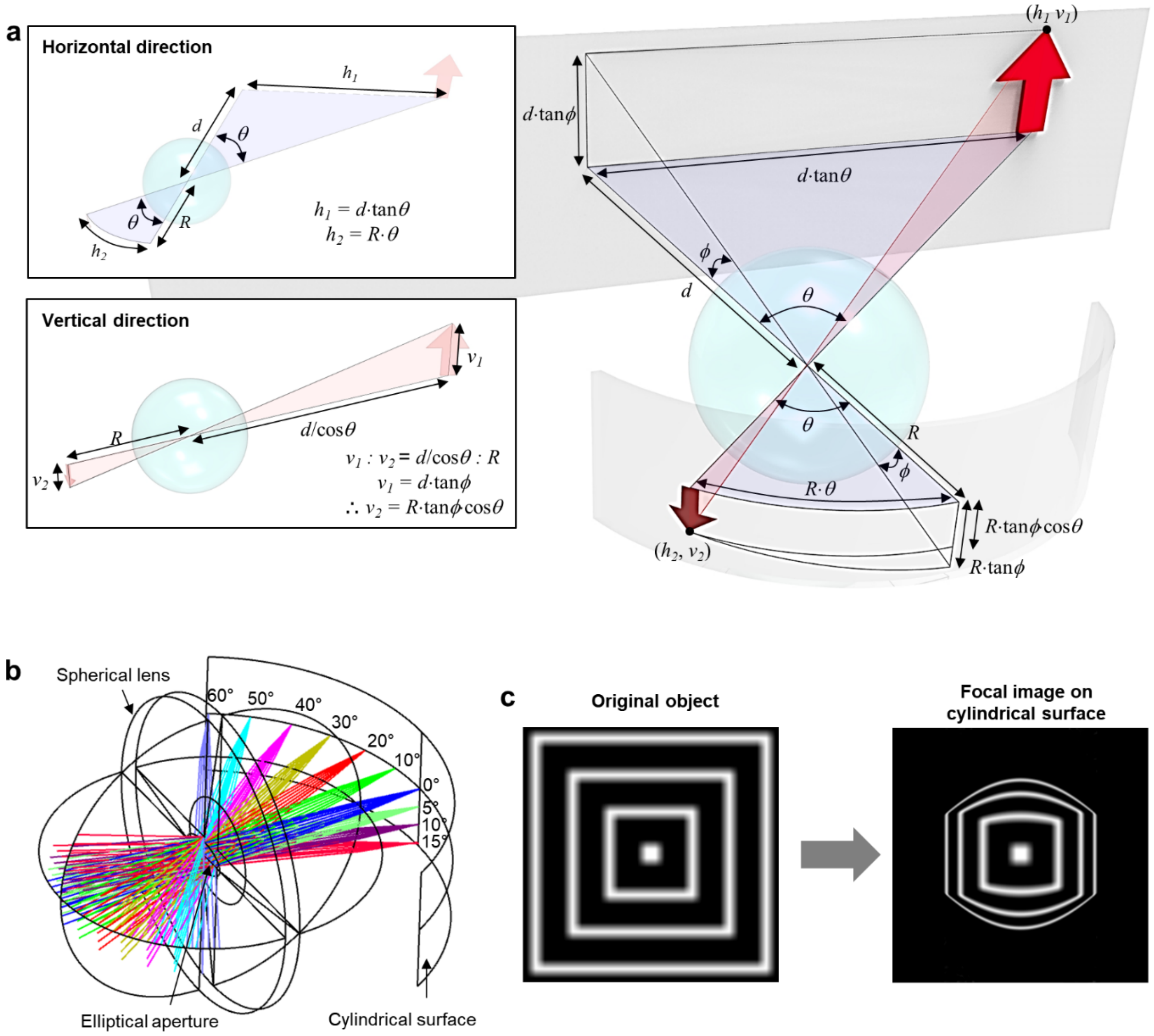

Supplementary Fig. 1 | Image distortion. a, Illustration of the ray destination point (h_2,v_2) on the cylindrical sensor surface corresponding to a ray origin point (h_1,v_1) on the object plane to show the geometry of the image distortion. **b**, Illustration of 3D ray tracing simulation. **c**, The image of a wide FoV grid object (FoV: –60° ~ 60°) (left) is distorted on the cylindrical sensor surface (right), obtained by the 3D ray tracing simulation.

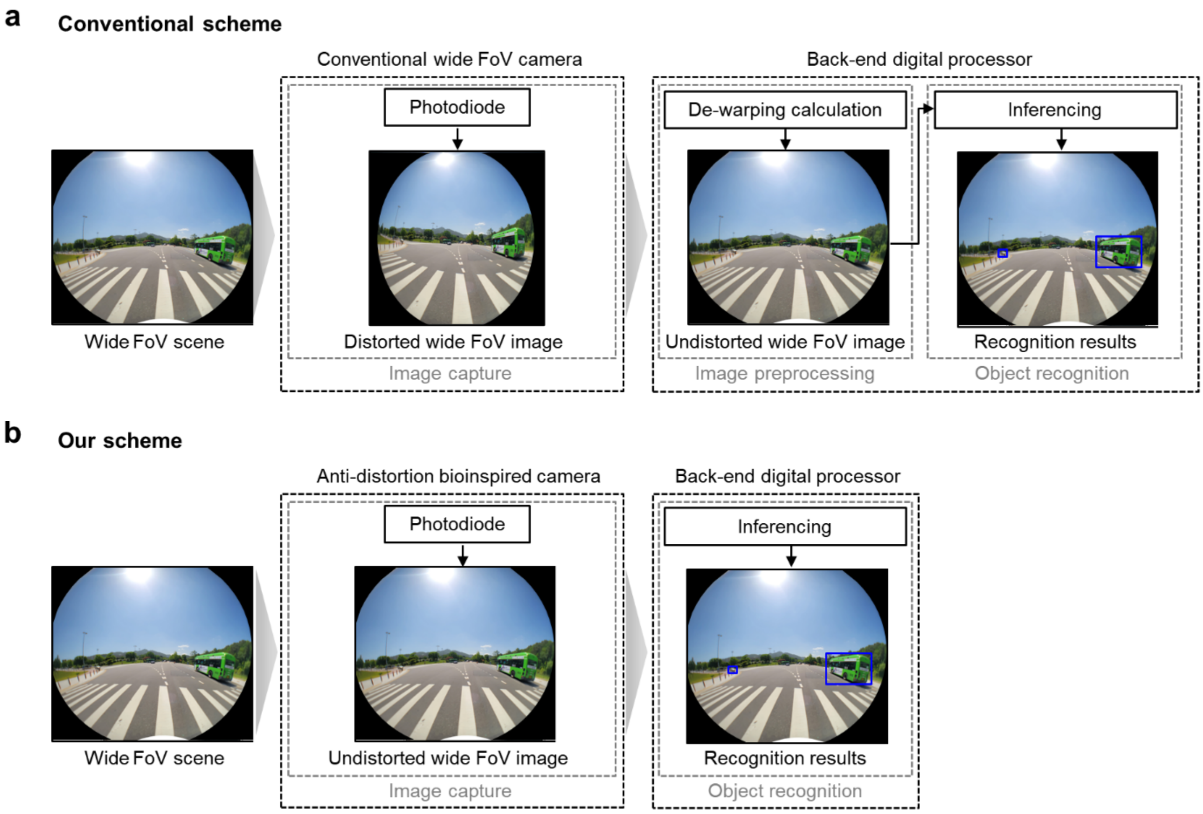

Supplementary Fig. 2 | Workflow difference for image distortion correction between conventional and our schemes. a, In the conventional scheme, a distorted image captured by the front-end image sensor is first sent to the back-end digital processor, wherein the distortion is first corrected by image de-warping software and then object recognition is performed. **b**, In our scheme, the front-end image sensor with the anti-image distortion capability captures an undistorted image. This would then be sent to the back-end digital processor, which would perform object recognition with no de-warping software. Thus our scheme is free of power consumption and processing time associated with the de-warping calculation. **Additional discussion.** In the context of the conventional image processing scheme (part **a**), object recognition might be able to be done with no de-warping calculation but with a more advanced—more computationally intensive—neural network that may recognize distorted images well, but this scheme would increase power consumption and processing time associated with the neural network inferencing, while removing those associated with the de-warping calculation. Which of the two schemes—no de-warping calculation with more computationally intensive neural network vs. de-warping calculation with less computationally burdensome neural network—would win depends on the details, but either scheme relying on the back-end digital processor for handling the image distortion would be worse in power consumption and processing time than our scheme (part **b**) where image recognition can be done with structurally-enabled anti-distortion image capture with less computationally burdensome neural network.

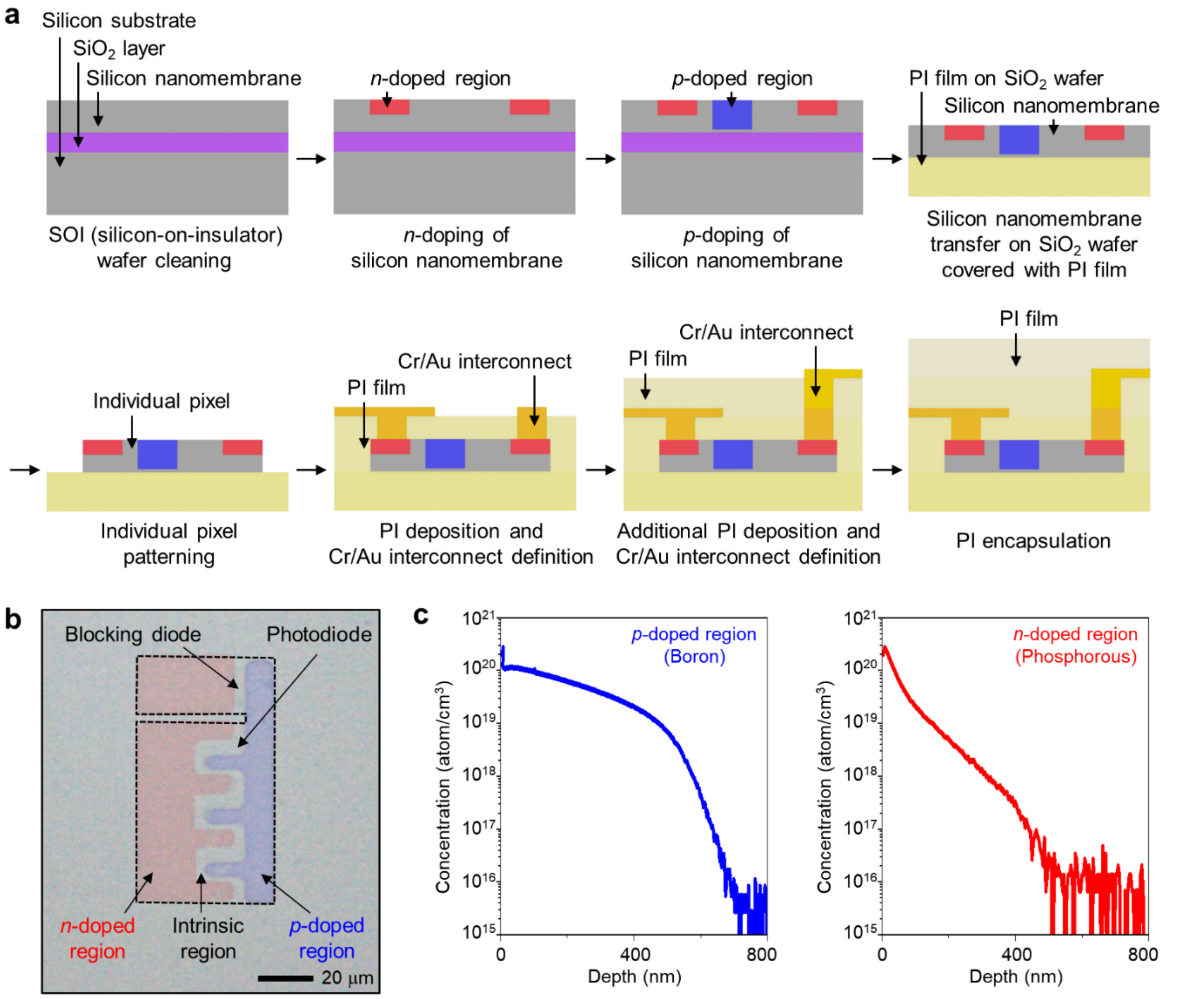

Supplementary Fig. 3 | Fabrication of the silicon photodiode array. a, Schematic illustration of the fabrication steps for the silicon photodiode array before transfer to the cylindrical PDMS substrate. **b**, Micrograph showing a single photo-pixel in the photodiode array, with the *n*- and *p*-doped regions shown in red and blue. **c**, Dopant concentration vs. depth for the *p*- and *n*-doped regions in a representative pixel, obtained using dynamic secondary ion mass spectroscopy (IMS 7f-Auto, CAMECA).

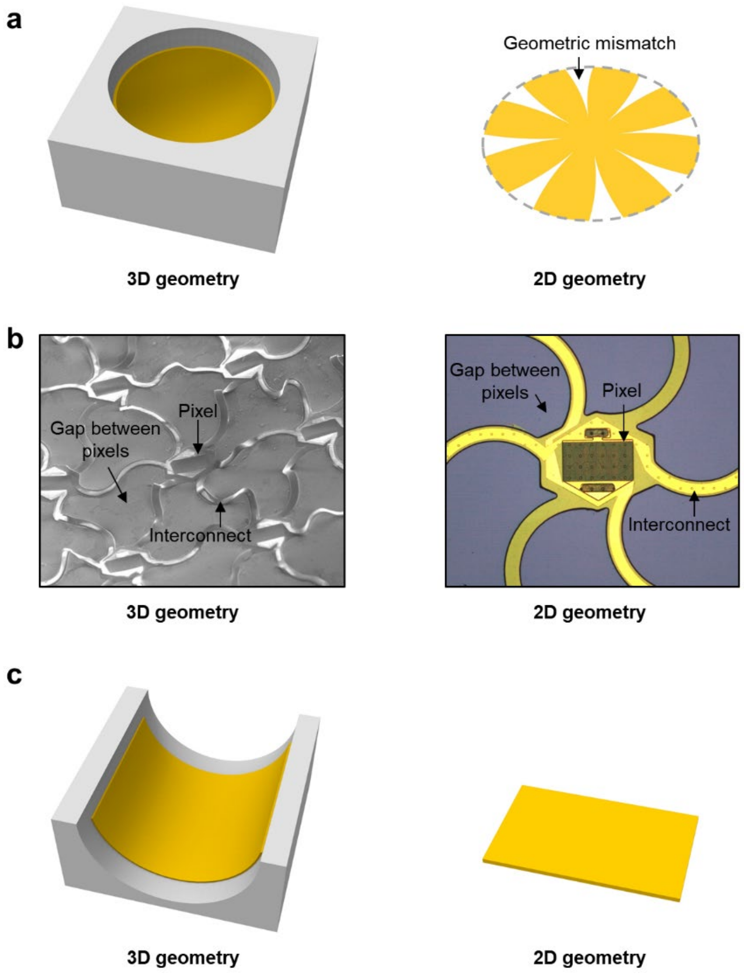

Supplementary Fig. 4 | Transfer of planar flexible electronics onto curved surface. a, If a flexible substrate on a three-dimensional (3D) hemispherical surface is transferred onto a two-dimensional (2D) plane, it does not fill a full disk but leaves empty spaces. Thus, conversely, if a full planar disk substrate is transferred onto a hemispherical surface, wrinkles will be formed. **b**, Scanning electron microscope and optical images of interconnected silicon photodiodes transferred onto a hemispherical surface. Here to mitigate wrinkle formation, the substrate is mostly empty, with the interconnects provided in the serpentine form. With such measure, pixels cannot be densely populated. **c**, A flexible substrate on a 3D cylindrical surface is transferred onto a 2D plane without creating empty spaces. Thus, conversely, a planar substrate can be seamlessly transferred onto the cylindrical surface without wrinkle formation.

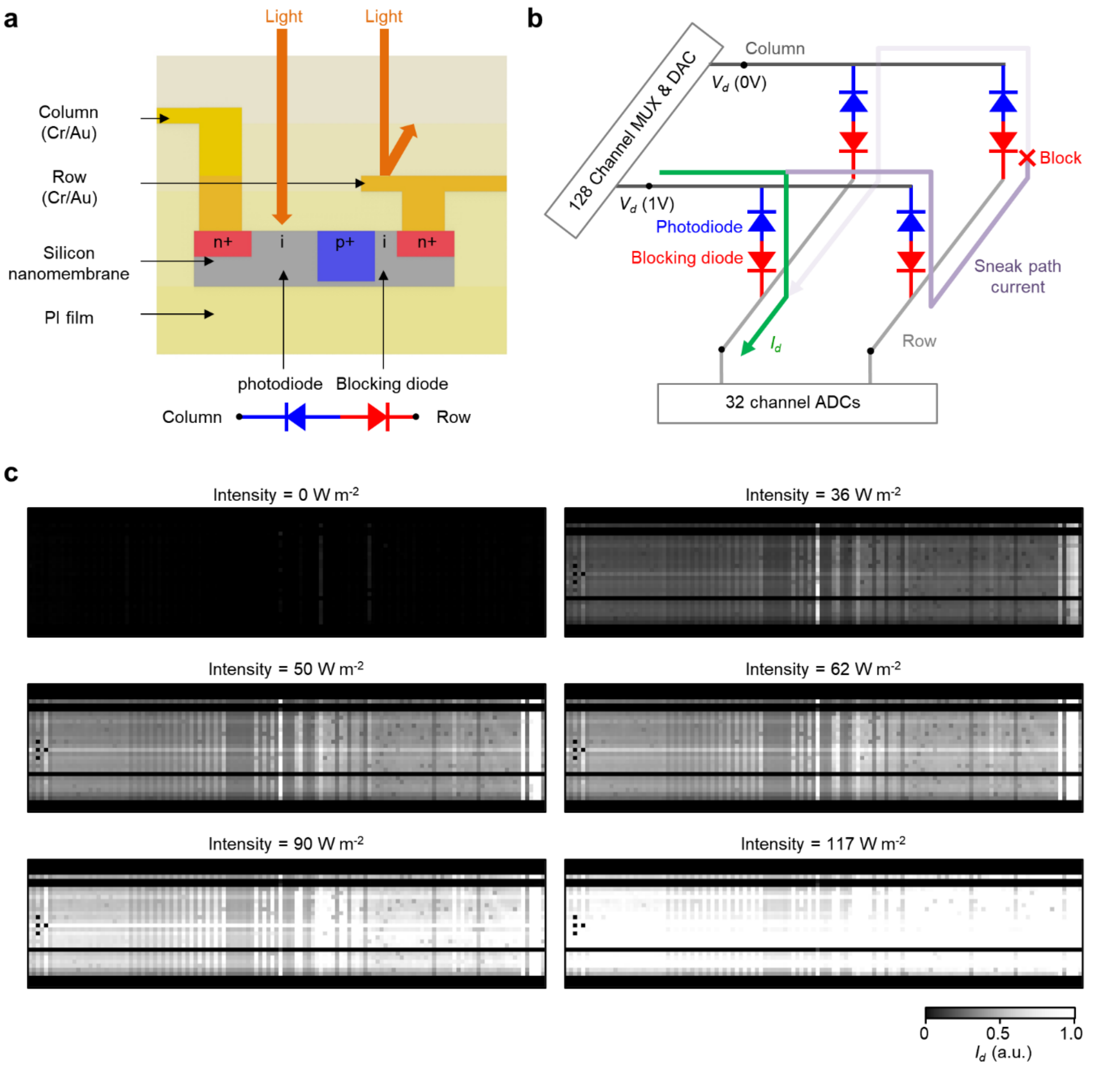

Supplementary Fig. 5 | Pixel cross section, sneak path current blocking, and array-wide measurement of photocurrents. a, Illustration of the pixel cross section. **b**, Illustration of the sneak path current blocking in the pixel array. **c**, Measured distribution of photocurrents across the 128 × 32 pixels on the cylindrical surface upon uniform light irradiation with varying intensities (*i.e.*, 0, 36, 50, 62, 90, and 117 W m^{-2}). Chronologically, these measurements were done after all the main imaging of Figs. 4 and 5 of the main text; what might be row line disconnections to the electronics seem to have been caused between the main imaging of Figs. 4 and 5 of the main text and these measurements here, leading to some horizontal dead lines.

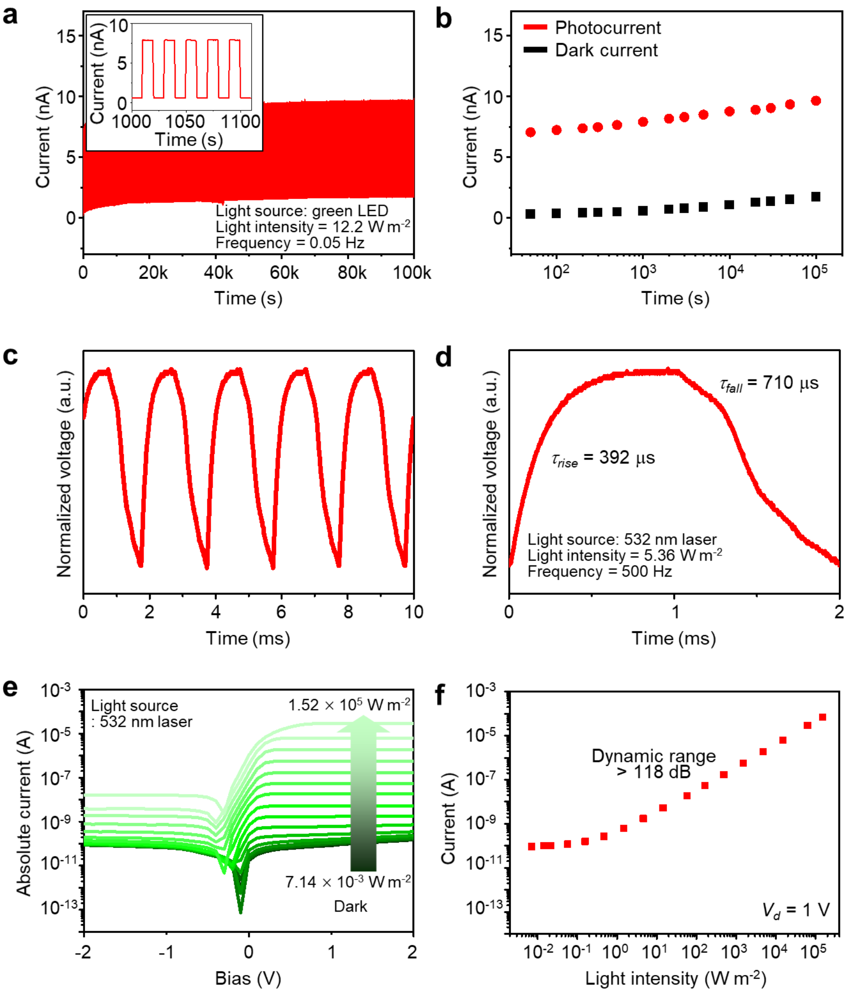

Supplementary Fig. 6 | Additional photoresponse characterization of a single pixel. a, Long-term photoresponse of a single pixel under illumination by a green LED (intensity: 12.2 W m^{-2}) pulsed at 0.05 Hz. Inset is the magnification between 1,000 s and 1,100 s. **b**, Photocurrents and dark currents of the pixel, extracted from part **a**. **c**,**d**, Photoresponse of the pixel (part **c**) and its magnified view (part **d**) under illumination by a 532-nm laser diode (DJ532-40, Thorlabs; intensity: 5.36 W m^{-2}) pulsed at 500 Hz. Here the photocurrent is converted to a voltage by a current preamplifier (SR570, Stanford Research Systems), and the voltage read by an oscilloscope (TDS2024, Tektronix) is plotted after normalization. The rise time (τ_{rise}), defined as a rise duration from 10% to 90% of the peak value, is 392 μs. The similarly-defined fall time (τ_{fall}) is 710 μs. **e**, Measured current vs. bias voltage of the pixel, under illumination by the 532-nm laser diode at intensities of 0, 7.14×10^{-3}, 1.43×10^{-2}, 2.14×10^{-2}, 5.71×10^{-2}, 1.57×10^{-1}, 4.71×10^{-1}, 1.47, 4.63, 1.45×10, 5.82×10, 1.66×10^{2}, 4.86×10^{2}, 1.52×10^{3}, 4.79×10^{3}, 1.51×10^{4}, 6.36×10^{4}, and 1.52×10^{5} W m^{-2}. To plot the current in the log scale, we use absolute values for the current data here. **f**, Measured current of the pixel at $V_d = 1$ V at various light intensities, extracted from part **e**. The output dynamic range exceeds 118 dB.

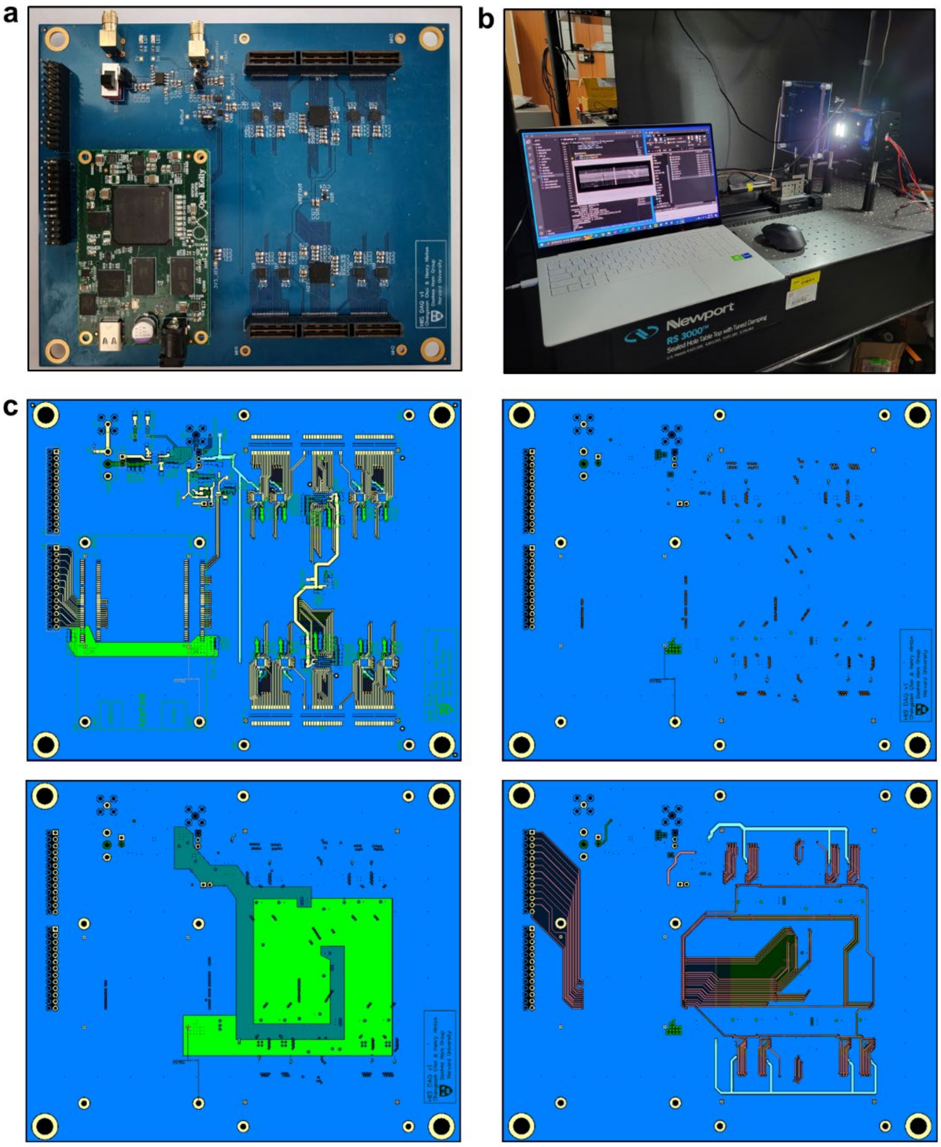

Supplementary Fig. 7 | Image acquisition electronics and setup. a, Photograph of the 4-layer printed circuit board (PCB), which hosts the electronics to read out the photocurrents from the 128 × 32 pixels. **b**, Photograph of the experimental setup used for imaging experiments. **c**, Layout of each layer in the 4-layer PCB.

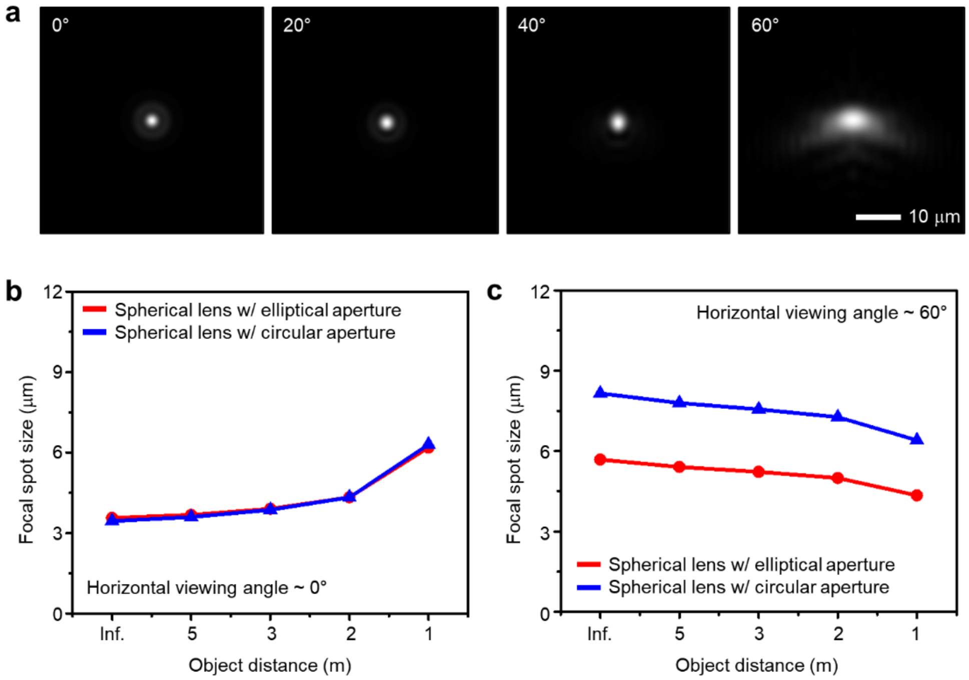

Supplementary Fig. 8 | Simulated focal spot sizes of our anti-distortion bioinspired camera for elliptical and circular aperture. a, Simulated point spreading function for various horizontal viewing angles, assuming a circular aperture (diameter: 1.88 mm). **b**,**c**, Simulated focal spot size as a function of the object distance at the horizontal viewing angle of 0° (part **b**) and 60° (part **c**) for the elliptical aperture we actually use and the 1.88-mm diameter circular aperture. The focal spot size for the elliptical aperture is the same as, or smaller than, that for the circular aperture across the horizontal FoV.

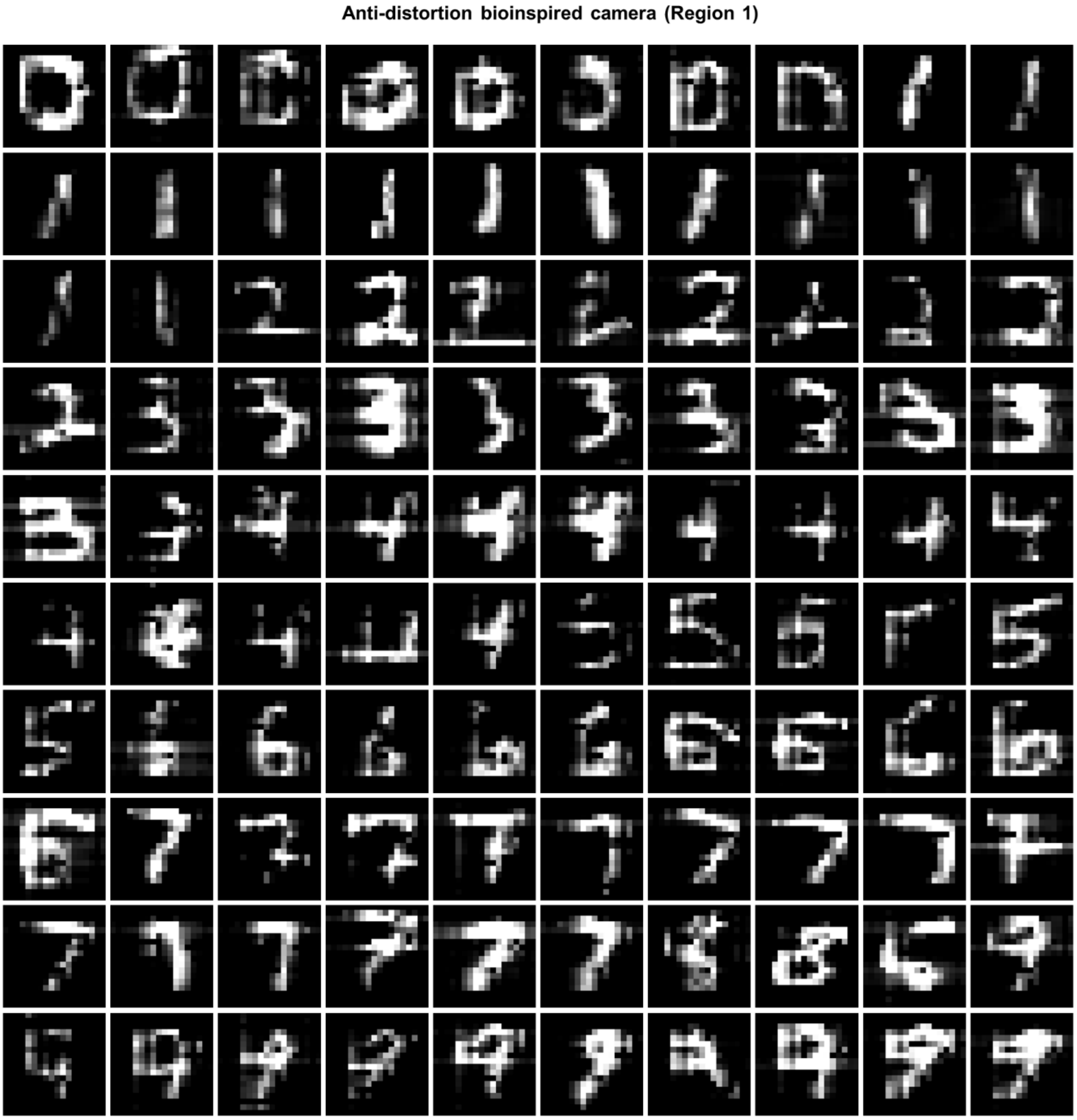

Supplementary Fig. 9 | Handwritten digit images captured in Region 1 of the cylindrical image sensor. This figure displays 100 MNIST handwritten digit images that are captured by Region 1 of our anti-distortion bioinspired camera.

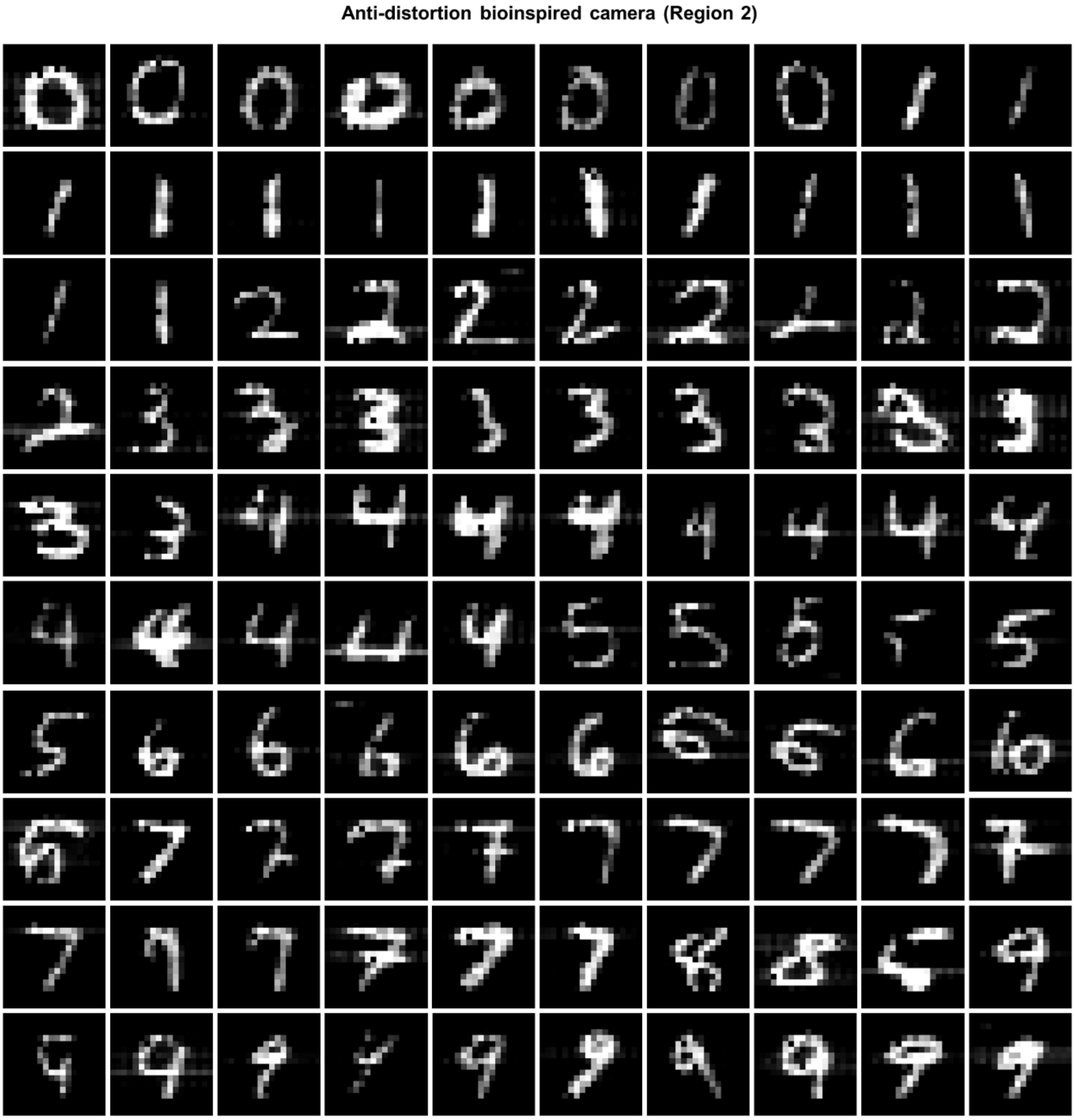

Supplementary Fig. 10 | Handwritten digit images captured in Region 2 of the cylindrical image sensor. This figure displays 100 MNIST handwritten digit images that are captured by Region 2 of our anti-distortion bioinspired camera.

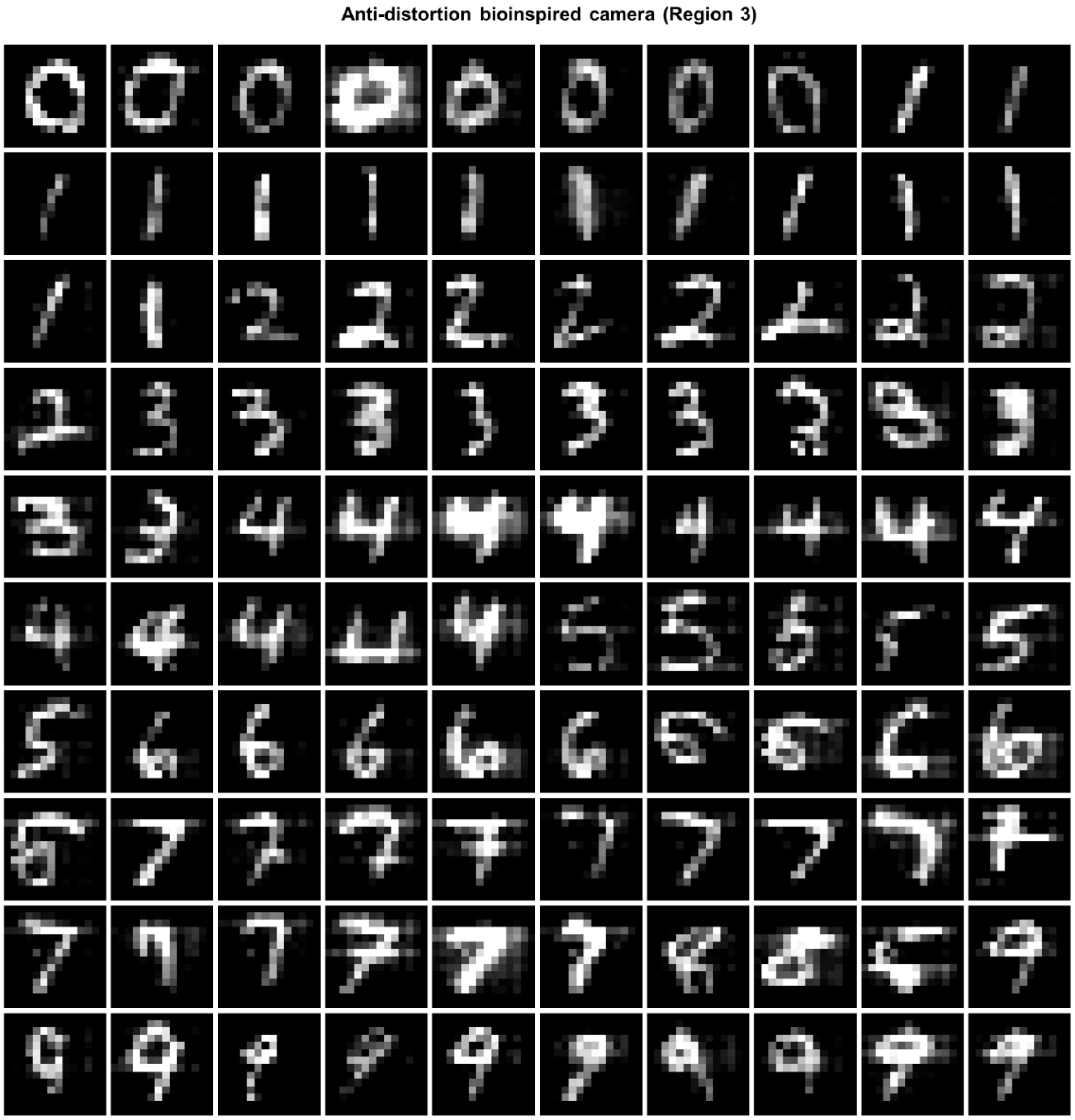

Supplementary Fig. 11 | Handwritten digit images captured in Region 3 of the cylindrical image sensor. This figure displays 100 MNIST handwritten digit images that are captured by Region 3 of our anti-distortion bioinspired camera.

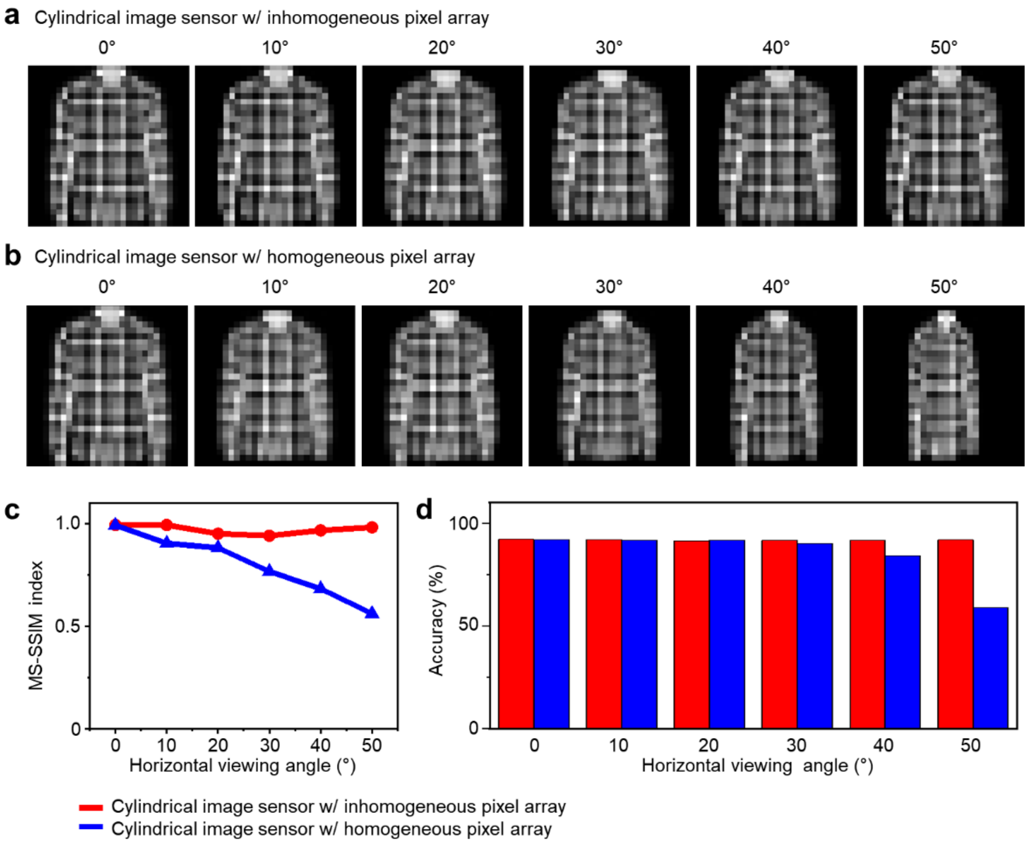

Supplementary Fig. 12 | Simulated acquisition of fashion image by the inhomogeneous and homogeneous pixel array. a,**b**, Simulated fashion images—using the 3D ray tracing simulation (Methods)—acquired by the cylindrical image sensor with the inhomogeneous (part **a**) and homogeneous (part **b**) pixel array at various horizontal viewing angles. One object image selected from the fashion-MNIST database is used for this simulation work. The homogeneous pixel array exhibits appreciable image distortion at larger horizontal viewing angles, while the inhomogeneous pixel array does not. **c**, Multi-scale structural similarity (MS-SSIM) index of the simulated fashion images in reference to the original object image. In the case of the homogenous pixel array, the index is substantially decreased from 1 with an increasing horizontal viewing angle due to distortion. **d**, Recognition accuracies of the simulated fashion images in CNN inferencing (Methods). The images acquired by the inhomogeneous pixel array with negligible distortion are recognized with an accuracy larger than ~91% across all horizontal viewing angles, whereas the recognition accuracy of the images acquired by the homogeneous pixel array decreases to ~59% at the large horizontal viewing angle due to distortion.

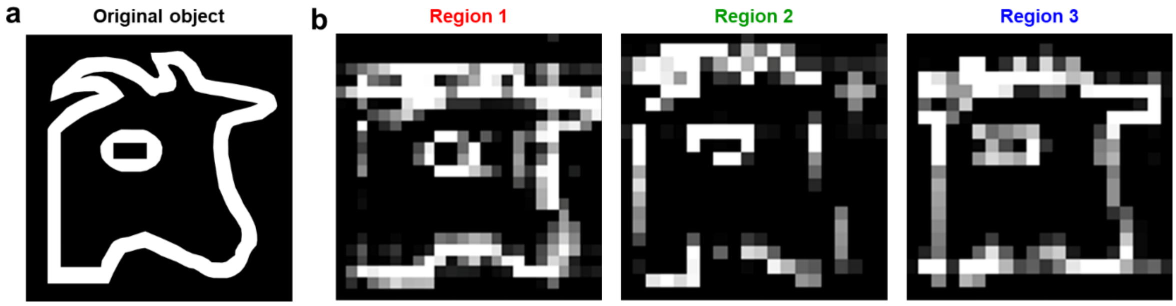

Supplementary Fig. 13 | Goat images captured by our anti-distortion bioinspired camera. a, An original goat image. **b**, Its capture by our camera in Regions 1, 2, and 3. No appreciable distortion is observed.

Anti-distortion bioinspired camera (F/# = 4.18)						
**Surface**	**Radius (mm)**	**Thickness (mm)**	**Material**	**RI**	**Abbe number**	**Semi-diameter (mm)**
OBJECT	Infinity	Infinity	-	-	-	Infinity
1	5.000	5.000	BK7	1.52	64.2	5.000
STOP	Infinity	0.500	NOA 61	1.56	42.4	1.000
4	Infinity	5.000	BK7	1.52	64.2	1.700
5	--5.000	2.087	-	-	-	5.000
IMAGE	-7.500	-	-	-	-	6.500

Supplementary Table 1 | Camera construct details. Geometric and material properties of each component used in our anti-distortion bioinspired camera.

Specification of bioinspired cameras							
Bioinspired camera	Shape of image sensor	Number of pixels	Interconnect design	Optical system	Pixel distribution	Distortion correction	Ref
Artificial human eye	Spherical	256	Pop-up	Single lens (Plano-convex)	Homogeneous	N/A	[S1]
Shape-tunable electronic eye	Spherical	256	Serpentine	Single lens (Plano-convex)	Homogeneous	N/A	[S2]
Artificial aquatic vision	Spherical	351	Serpentine	Single lens (Spherical)	Homogeneous	N/A	[S3]
Neuromorphic artificial eye	Spherical	31	Mesh	Single lens (Plano-convex)	Homogeneous	N/A	[S4]
Electrochemical biomimetic eye	Spherical	100	N/A	Single lens (N/A)	Homogeneous	N/A	[S5]
Hemispherical electronic eye	Spherical	676	Kirigami	Single lens (Plano-convex)	Homogeneous	N/A	[S6]
Shape-adaptive imager	Spherical	1024	Kirigami	Single lens (Plano-convex)	Homogeneous	N/A	[S7]
Artificial cuttlefish eye	Cylindrical	380	Straight line	Single lens (Spherical)	Inhomogeneous	N/A	[S8]
Anti-distortion bioinspired camera	Cylindrical	4096	Straight line	Single lens (Spherical)	Inhomogeneous	Configurational in-sensor computing	This work

Supplementary Table 2 | Comparison of the specifications of our bioinspired camera vs. other bioinspired cameras. Our camera not only has the highest number of pixels but also uniquely features the anti-image distortion capability. Ref. S8 also reports inhomogeneous distribution of pixels, but this is to concentrate pixels more densely at the center of the sensor surface to achieve higher resolution therein, not to correct image distortion.

Supplementary References

S1. Ko, H. C. *et al.* A hemispherical electronic eye camera based on compressible silicon optoelectronics. *Nature* **454**, 748–753 (2008).

S2. Jung, I. *et al.* Dynamically tunable hemispherical electronic eye camera system with adjustable zoom capability. *Proc. Natl. Acad. Sci. U.S.A.* **108**, 1788–1793 (2011).

S3. Kim, M., *et al.* An aquatic-vision-inspired camera based on a monocentric lens and a silicon nanorod photodiode array. *Nat. Electron.* **3**, 546-553 (2020).

S4. Choi, C. *et al.* Curved neuromorphic image sensor array using MoS_2-organic heterostructure inspired by human visual recognition system. *Nat. Commun.* **11**, 5934 (2020).

S5. Gu, L. *et al.* A biomimetic eye with a hemispherical perovskite nanowire array retina. *Nature* **581**, 278–282 (2020).

S6. Zhang, K., *et al.* Origami silicon optoelectronics for hemispherical electronic eye systems. *Nat. Commun.* **8**, 1782 (2017).

S7. Rao, Z. *et al.* Curvy, shape-adaptive imagers based on printed optoelectronic pixels with a kirigami design. *Nat. Electron.* **4**, 513-521 (2021).

S8. M, Kim. *et al.* Cuttlefish eye–inspired artificial vision for high-quality imaging under uneven illumination conditions. *Sci. Robot.* **8**, eade4698 (2023).
