# Supplementary material for: Anti-distortion bioinspired camera with an inhomogeneous photo-pixel array
Source: Nat Commun. 2024 Jul 17;15:6021. doi: 10.1038/s41467-024-50271-7 (PMC11255341; doi:10.1038/s41467-024-50271-7)
Supplement: Supplementary file 2 — Peer Review File [file 41467_2024_50271_MOESM2_ESM.pdf]

nature portfolio

Peer Review File

Anti-distortion bioinspired camera with an inhomogeneous photo-pixel array

Open Access This file is licensed under a Creative Commons Attribution 4.0 International License, which permits use, sharing, adaptation, distribution and reproduction in any medium or format, as long as you give appropriate credit to the original author(s) and the source, provide a link to the Creative Commons license, and indicate if changes were made. In the cases where the authors are anonymous, such as is the case for the reports of anonymous peer reviewers, author attribution should be to 'Anonymous Referee' followed by a clear attribution to the source work. The images or other third party material in this file are included in the article's Creative Commons license, unless indicated otherwise in a credit line to the material. If material is not included in the article's Creative Commons license and your intended use is not permitted by statutory regulation or exceeds the permitted use, you will need to obtain permission directly from the copyright holder. To view a copy of this license, visit http://creativecommons.org/licenses/by/4.0/.

REVIEWER COMMENTS

Reviewer #1 (Remarks to the Author):

This paper presents an anti-distortion single-lens camera constructed by a 128×32 Si photodiodes array, which is assigned creatively in an inhomogeneous pattern as same manner as the light with nonlinearly scaled that arrives on the curved sensor surface. This approach is available to capture undistorted images across a 120° horizontal field of view, enhancing the accuracy of image recognition processed by neural network. Despite there are several missing information that need to be addressed, the original paper is well organized and clear in its theme. Therefore, the manuscript may be considered for publication in this journal with major revisions on addressing points outlined below.

1. Authors should provide a detailed process flow chart to illustrate how to pattern doping region as demonstrated device structure in Supplementary Fig. 4a with spin-coated process.

2. Despite the fact that authors claimed N/P regions of silicon photodiode were defined sequentially by SOD solutions, there is no relevant characterization to prove doping location, depth, concentration or other key parameters.

3. In figure 3d, the authors demonstrated the current vs bias voltage of image sensor under light illumination with various illumination intensities. Why is the photocurrent only a few nanoamps for the silicon-based image sensor?

4. There are limited optical and electrical measurements to display the performance of flexible photodiode. The reviewer believes that putting more tests on aspects of volt-ampere characteristic of the p-i-n photodiode, endurance analysis, photo-response speed, dynamic range, etc. can significantly improve the quality of the manuscript.

5. Why are images acquired at region 1 (with a smaller horizontal viewing, 0-30°) recognized with even a lower average accuracy than images acquired at region 2 (with a larger horizontal viewing, 30-45°)? Please comment.

6. The spatial distribution of photocurrent from sensor array in Supplementary Fig. 4 seems to display a higher device-to-device variation.

7. Please clearly benchmark the work against existing similar or more advanced works

Reviewer #2 (Remarks to the Author):

This work proposed an inhomogeneous photodiode array for anti-distortion at the front sensor end. Overall, the work is valuable for advanced curved image sensors with more computing capabilities. However, the following concerns have to be addressed.
1. How robust is the system for anti-distortion when changing the input images with a more complex pattern or adding more noise? Will the determined boundary in the current inhomogeneous array also apply to different input images? The authors should demonstrate the feasibility and robustness of this array under different application scenarios.
2. The authors should provide more details regarding the novelty of this work instead of the engineering efforts as the inhomogeneous approaches have been already reported in the previous report.
3. The authors should compare this system with the previously reported hardware for anti-distortion regarding power consumption, chip area, speed, anti-distortion robustness under different application scenarios, etc.
4. The comparison schematics of the conventional hardware strategy or architecture for anti-distortion and the proposed method should be provided for better understanding.
5. The details of the neural network should be supplemented. Suppose a more robust neural network is used for accuracy comparison or the input images are very noisy or with a much larger size. Does the distortion correction really affect the network accuracies to a large degree?

<u>Point-by-Point Response to Reviewer 1</u>

Comment #0: *"This paper presents an anti-distortion single-lens camera constructed by a 128×32 Si photodiodes array, which is assigned creatively in an inhomogeneous pattern as same manner as the light with nonlinearly scaled that arrives on the curved sensor surface. This approach is available to capture undistorted images across a 120° horizontal field of view, enhancing the accuracy of image recognition processed by neural network. Despite there are several missing information that need to be addressed, the original paper is well organized and clear in its theme. Therefore, the manuscript may be considered for publication in this journal with major revisions on addressing points outlined below."*

Response: We thank Reviewer #1 for the encouraging comment and valuable feedback on our manuscript. We have addressed all of the reviewer comments in this revision, as we detail below.

Comment #1: *"Authors should provide a detailed process flow chart to illustrate how to pattern doping region as demonstrated device structure in Supplementary Fig. 4a with spin-coated process."*

Response: Following this advice, this revision provides more details of the device fabrication in Methods and adds a new Supplementary Fig. 3 to show the fabrication steps as follows:

> [**Methods**] "Here we detail the fabrication of the cylindrical image sensor, in connection with Supplementary Fig. 3. A 1.25 μm thick silicon nanomembrane on an SOI wafer (SOITEC) is first spin-coated with an *n*-type spin-on-dopant (SOD) solution (P509, Filmtronics) and annealed at 975 °C for 12 minutes to create *n*-doped regions. Then, the silicon nanomembrane is spin-coated with a *p*-type SOD solution (B153, Filmtronics) and annealed at 975 °C for 30 minutes to create *p*-doped regions. Note that before each spin coating, a 300-nm SiO_2 layer is deposited on the silicon nanomembrane using plasma-enhanced chemical vapor deposition (PECVD) and is subsequently patterned to define spin-coating regions, and after each spin coating and annealing, the SiO_2 layer and residual SOD solution are removed. The doping profiles are analyzed using dynamic secondary ion mass spectroscopy (IMS 7f-Auto, CAMECA; Supplementary Fig. 3c). The doped silicon nanomembrane is then separated from the SOI wafer and is transferred to a SiO_2 wafer covered with a PI film, and individual pixels are patterned using photolithography and reactive ion etching. An additional PI film is then spin-coated, cured, and patterned to form holes, which are subsequently filled with Cr/Au that is deposited with thermal evaporation to form vertical interconnects. The resulting Cr/Au layer is ~ 10/100 nm thick, which is patterned using photolithography and wet etching also to define lateral interconnects. These steps were repeated to form two layers of lateral Cr/Au interconnects. An additional PI film is then added for encapsulation. The resulting silicon photodiode array is then transferred onto the cylindrical PDMS substrate with a radius of curvature of 7.5 mm using a wafer-soluble tape (3M Corp.). This completes the fabrication of the cylindrical image sensor."
>
> **[Supplementary Information]**

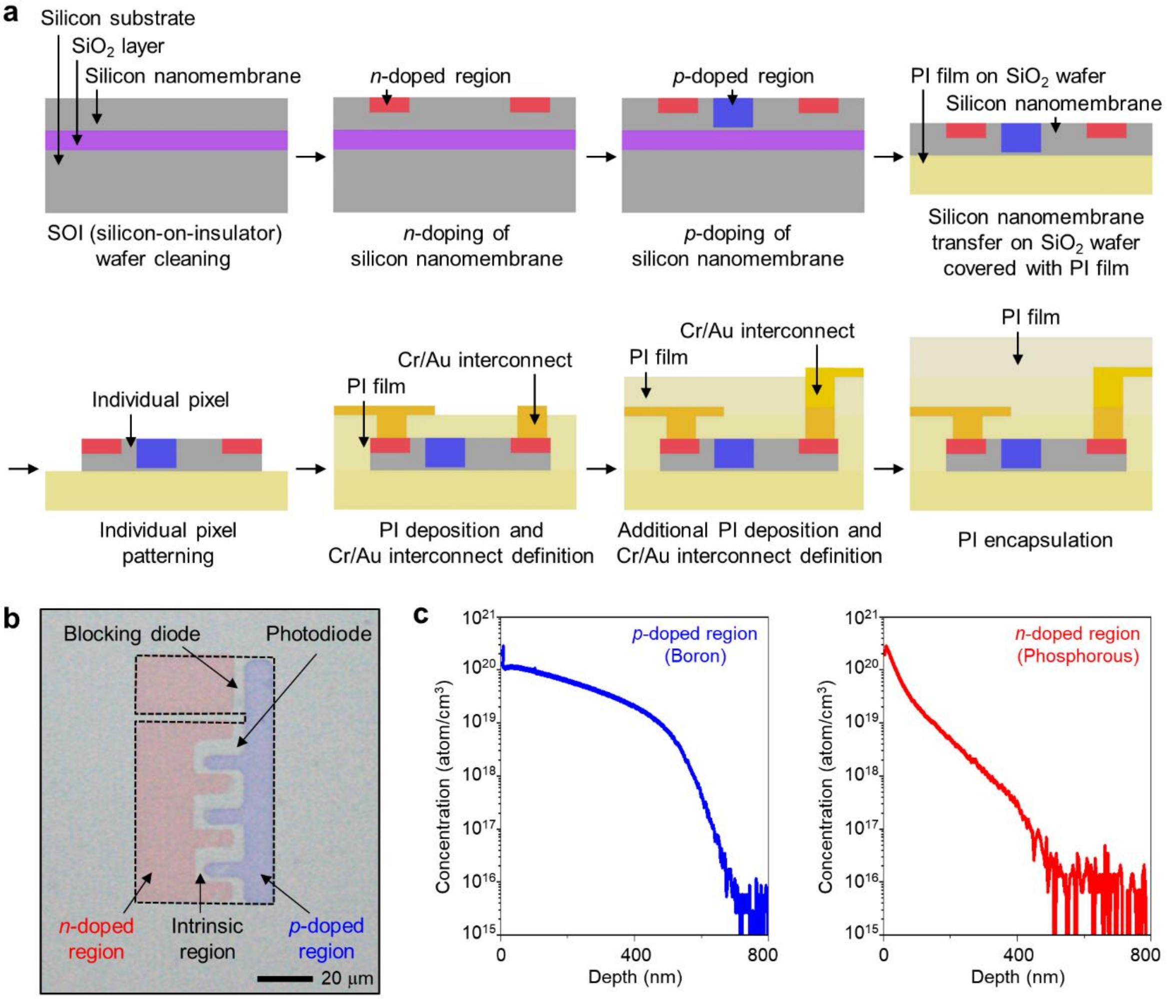

Supplementary Fig. 3 | Fabrication of the silicon photodiode array. a, Schematic illustration of the fabrication steps for the silicon photodiode array before transfer to the cylindrical PDMS substrate. **b**, Micrograph showing a single photo-pixel in the photodiode array, with the *n*- and *p*-doped regions shown in red and blue. **c**, Dopant concentration vs. depth for the *p*- and *n*-doped regions in a representative pixel, obtained using dynamic secondary ion mass spectroscopy (IMS 7f-Auto, CAMECA).

Comment #2: *"Despite the fact that authors claimed N/P regions of silicon photodiode were defined sequentially by SOD solutions, there is no relevant characterization to prove doping location, depth, concentration or other key parameters."*

Response: Following this advice, we have used dynamic secondary ion mass spectroscopy to obtain dopant concentration vs. depth for the *p*- and *n*-doped regions in a representative pixel, which we present in new Supplementary Fig. 3 (part **c**), as shown above in **Response to Reviewer 1, Comment #1**. Also in Methods, we refer to this new Supplementary Fig. 3c as follows: "The doping profiles are analyzed using dynamic secondary ion mass spectroscopy (IMS 7f-Auto, CAMECA; Supplementary Fig. 3c)." Finally, note that the micrograph of the new Supplementary Fig. 3 (part **b**) also explicitly shows the *n*- and *p*-doped regions.

Comment #3: *"In figure 3d, the authors demonstrated the current vs bias voltage of image sensor under light illumination with various illumination intensities. Why is the photocurrent only a few nanoamps for the silicon-based image sensor?"*

Response: We thank the reviewer for this comment. We realize that the light intensity values in Fig. 3d in our original submission were underestimated (*i.e.*, the photo responsivity was over-estimated), and for this revision, we have newly measured the current vs. bias voltage of a pixel under light illumination with various intensities (now with better calibration), and have updated Fig. 3d as follows.

[Main text]

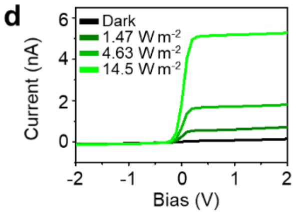

"**Fig. 3d**, Measured current vs. bias voltage of a representative pixel, under light illumination with various intensities (light source: 532-nm laser diode). Its responsivity is 0.10 A W^{-1} for an intensity of 1.47 W m^{-2}. The results for a wide range of intensities, spanning from 7.14×10^{-3} W m^{-2} to 1.52×10^{5} W m^{-2}, are shown in Supplementary Fig. 6e."

Furthermore, as detailed in the updated caption above, the new measurement exhibits a responsivity of 0.10 A W^{-1} (wavelength: 532 nm, intensity: 1.47 W m^{-2}) (the responsivity in our original submission with the underestimated light intensity was substantially higher). This is not too significantly lower than the typical responsivity of silicon photodiodes at the same wavelength, typically ranging from 0.15 to 0.30 A W^{-1}.

Comment #4: *"There are limited optical and electrical measurements to display the performance of flexible photodiode. The reviewer believes that putting more tests on aspects of volt-ampere characteristic of the p-i-n photodiode, endurance analysis, photo-response speed, dynamic range, etc. can significantly improve the quality of the manuscript."*

Response: Following this advice, we have measured more photo-response properties, including endurance, response time, and dynamic range, and present them in new Supplementary Fig. 6 as follows:

[Supplementary Information]

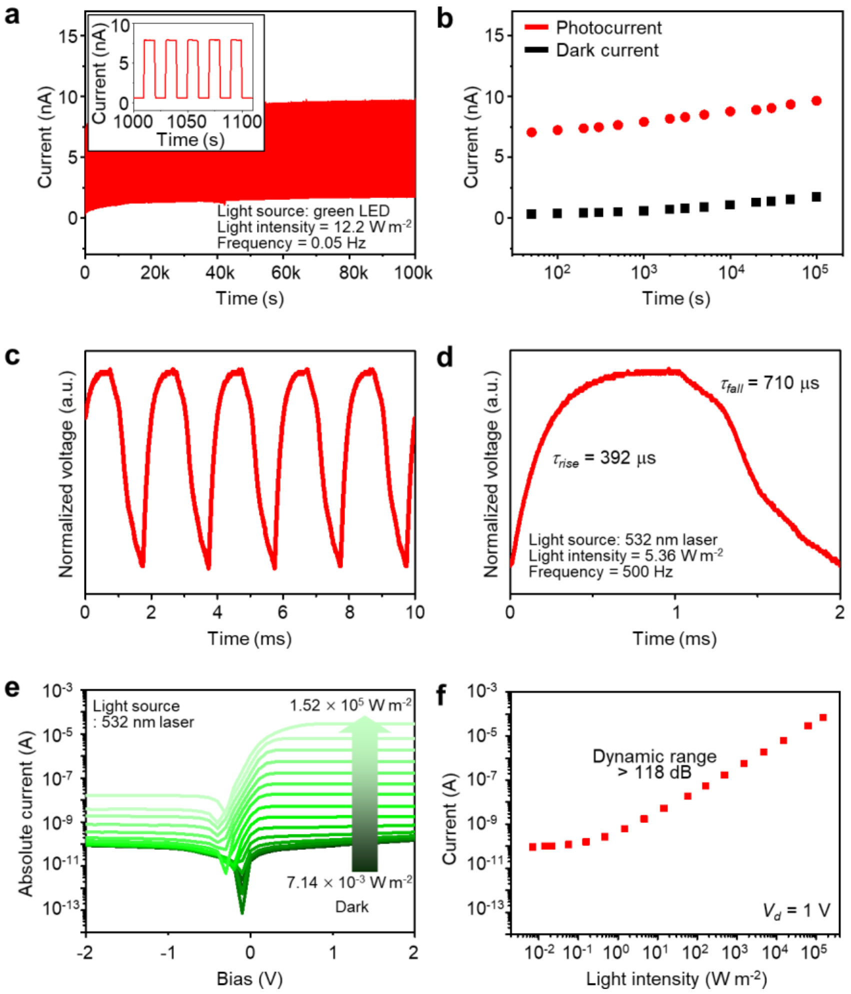

Supplementary Fig. 6 | Additional photoresponse characterization of a single pixel. a, Long-term photoresponse of a single pixel under illumination by a green LED (intensity: 12.2 W m^{-2}) pulsed at 0.05 Hz. Inset is the magnification between 1,000 s and 1,100 s. **b**, Photocurrents and dark currents of the pixel, extracted from part **a**. **c**,**d**, Photoresponse of the pixel (part **c**) and its magnified view (part **d**) under illumination by a 532-nm laser diode (DJ532-40, Thorlabs; intensity: 5.36 W m^{-2}) pulsed at 500 Hz. Here the photocurrent is converted to a voltage by a current preamplifier (SR570, Stanford Research Systems), and the voltage read by an oscilloscope (TDS2024, Tektronix) is plotted after normalization. The rise time (τ_{rise}), defined as a rise duration from 10% to 90% of the peak value, is 392 µs. The similarly-defined fall time (τ_{fall}) is 710 µs. **e**, Measured current vs. bias voltage of the pixel, under illumination by the 532-nm laser diode at intensities of 0, 7.14×10^{-3}, 1.43×10^{-2}, 2.14×10^{-2}, 5.71×10^{-2}, 1.57×10^{-1}, 4.71×10^{-1}, 1.47, 4.63, 1.45×10, 5.82×10, 1.66×10^{2}, 4.86×10^{2}, 1.52×10^{3}, 4.79×10^{3}, 1.51×10^{4}, 6.36×10^{4}, and 1.52×10^{5} W m^{-2}. To plot the current in the log scale, we use absolute values for the current data here. **f**, Measured current of the pixel at $V_d = 1$ V at various light intensities, extracted from part **e**. The output dynamic range exceeds 118 dB.

Comment #5: *Why are images acquired at region 1 (with a smaller horizontal viewing, 0-30°) recognized with even a lower average accuracy than images acquired at region 2 (with a larger horizontal viewing, 30-45°)? Please comment.*

Response: We thank Reviewer 1 for this important comment. We think that the images acquired in Region 2 result in higher recognition accuracy (93 %) than those in Region 1 (88 %), probably because Region 2 has relatively less pixel-to-pixel variations, as seen in Supplementary Fig. 5c (please pay attention to the right half of the pixel array). We have added this information in the main text as follows:

> **[Main text]** "We also note that the relatively higher recognition accuracy (93 %) for images acquired at Region 2 is due probably to the fact that Region 2 has relatively less pixel-to-pixel variations, as seen in Supplementary Fig. 5c."

Comment #6: *The spatial distribution of photocurrent from sensor array in Supplementary Fig. 4 seems to display a higher device-to-device variation.*

Response: As the reviewer correctly pointed out, there indeed is an appreciable amount of pixel-to-pixel variation as seen in that supplementary figure (which is now Supplementary Fig. 5), which arises from non-uniform doping of silicon photodiodes, non-uniform contact resistances, and possibly the fracturing of the silicon nanomembrane during the transfer process. These issues in our in-house fabrication may be mitigated in industry-standard fabrication. We have re-written the relevant passage in the main text as follows to articulate the issue:

> **[Main text]** "Each subfigure of Supplementary Fig. 5c shows photocurrents measured across the pixel array......... Not only do these measurements show the working of the electronics setup to read out the photocurrents across the pixel array, but they also reveal quite significant pixel-to-pixel variations, which may be attributed to non-uniform doping of silicon photodiodes, non-uniform contact resistances, and possible fracturing of the silicon nanomembrane during the transfer process. All of these are fabrication issues, which may be mitigated by transitioning to industry-standard fabrication from in-house fabrication."

Furthermore, chronologically, the measurements of Supplementary Fig. 5 (Supplementary Fig. 4 in the original submission) were done after all the main imaging of Figs. 4 and 5 of the main text; what might be row line disconnections to the electronics seem to have been caused between the main imaging of Figs. 4 and 5 of the main text and the measurements of Supplementary Fig. 5, leading to some horizontal dead lines in the latter. This revision now explicitly states this as follows in the caption of Supplementary Fig. 5:

> **[Supplementary Information]** "**Supplementary Fig. 5** ...... Chronologically, these measurements were done after all the main imaging of Figs. 4 and 5 of the main text; what might be row line disconnections to the electronics seem to have been caused between the main imaging of Figs. 4 and 5 of the main text and these measurements here, leading to some horizontal dead lines."

Comment #7: *Please clearly benchmark the work against existing similar or more advanced works*

Response: Following this advice, we compare our camera with other bioinspired cameras in the new Supplementary Table 2 as follows. As can be seen, our camera has the highest number of pixels and also uniquely features the anti-image distortion capability. While Ref. S8 also reports inhomogeneous distribution of pixels, this is to concentrate pixels more densely at the center of the sensor surface to achieve higher resolution therein, but not to correct image distortion.

> **[Supplementary Information]**

Specification of bioinspired cameras							
Bioinspired camera	Shape of image sensor	Number of pixels	Interconnect design	Optical system	Pixel distribution	Distortion correction	Ref
Artificial human eye	Spherical	256	Pop-up	Single lens (Plano-convex)	Homogeneous	N/A	[S1]
Shape-tunable electronic eye	Spherical	256	Serpentine	Single lens (Plano-convex)	Homogeneous	N/A	[S2]
Artificial aquatic vision	Spherical	351	Serpentine	Single lens (Spherical)	Homogeneous	N/A	[S3]
Neuromorphic artificial eye	Spherical	31	Mesh	Single lens (Plano-convex)	Homogeneous	N/A	[S4]
Electrochemical biomimetic eye	Spherical	100	N/A	Single lens (N/A)	Homogeneous	N/A	[S5]
Hemispherical electronic eye	Spherical	676	Kirigami	Single lens (Plano-convex)	Homogeneous	N/A	[S6]
Shape-adaptive imager	Spherical	1024	Kirigami	Single lens (Plano-convex)	Homogeneous	N/A	[S7]
Artificial cuttlefish eye	Cylindrical	380	Straight line	Single lens (Spherical)	Inhomogeneous	N/A	[S8]
Anti-distortion bioinspired camera	Cylindrical	4096	Straight line	Single lens (Spherical)	Inhomogeneous	Configurational in-sensor computing	This work

Supplementary Table 2 | Comparison of the specifications of our bioinspired camera vs. other bioinspired cameras. Our camera not only has the highest number of pixels but also uniquely features the anti-image distortion capability. Ref. S8 also reports inhomogeneous distribution of pixels, but this is to concentrate pixels more densely at the center of the sensor surface to achieve higher resolution therein, not to correct image distortion.

[Supplementary References]

S1. Ko, H. C. *et al.* A hemispherical electronic eye camera based on compressible silicon optoelectronics. *Nature* **454**, 748–753 (2008).
S2. Jung, I. *et al.* Dynamically tunable hemispherical electronic eye camera system with adjustable zoom capability. *Proc. Natl. Acad. Sci. U.S.A.* **108**, 1788–1793 (2011).
S3. Kim, M., *et al.* An aquatic-vision-inspired camera based on a monocentric lens and a silicon nanorod photodiode array. *Nat. Electron.* **3**, 546-553 (2020).
S4. Choi, C. *et al.* Curved neuromorphic image sensor array using MoS_2-organic heterostructure inspired by human visual recognition system. *Nat. Commun.* **11**, 5934 (2020).
S5. Gu, L. *et al.* A biomimetic eye with a hemispherical perovskite nanowire array retina. *Nature* **581**, 278–282 (2020).
S6. Zhang, K., *et al.* Origami silicon optoelectronics for hemispherical electronic eye systems. *Nat. Commun.* **8**, 1782 (2017).
S7. Rao, Z. *et al.* Curvy, shape-adaptive imagers based on printed optoelectronic pixels with a kirigami design. *Nat. Electron.* **4**, 513-521 (2021).
S8. M, Kim. *et al.* Cuttlefish eye–inspired artificial vision for high-quality imaging under uneven illumination conditions. *Sci. Robot.* **8**, eade4698 (2023).

Point-by-point Response to Reviewer 2

Comment #0: *This work proposed an inhomogeneous photodiode array for anti-distortion at the front sensor end. Overall, the work is valuable for advanced curved image sensors with more computing capabilities. However, the following concerns have to be addressed.*

Response: We thank Reviewer 2 for the encouragement and the valuable comments, which we have addressed in this revision, as we detail below.

Comment #1: *How robust is the system for anti-distortion when changing the input images with a more complex pattern or adding more noise? Will the determined boundary in the current inhomogeneous array also apply to different input images? The authors should demonstrate the feasibility and robustness of this array under different application scenarios.*

Response: To address, in addition to existing Supplementary Fig. 12 where we simulated fashion images captured by the inhomogeneous and homogeneous pixel arrays, with the former demonstrating no appreciable distortion, we now provide new Supplementary Fig. 13 as below, where we experimentally capture goat images at Regions 1, 2, and 3 with the inhomogeneous pixel array to show again anti-image distortion:

[Supplementary Information]

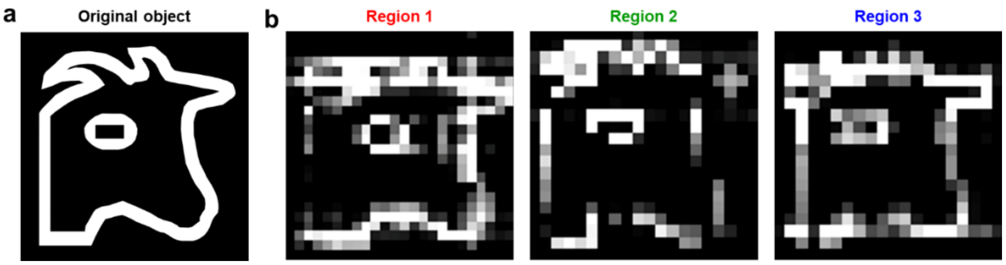

Supplementary Fig. 13 | Goat images captured by our anti-distortion bioinspired camera. a, An original goat image. **b**, Its capture by our camera in Regions 1, 2, and 3. No appreciable distortion is observed.

As for noise, silicon photodiodes, whether homogeneously distributed or not, have no particular noise suppression ability, and denoising in the front-end sensor could be a future research topic. Finally, the boundary of the FoV of our camera (horizontally ± 60° and vertically ± 15°) is independent of the image pattern.

Comment #2: *The authors should provide more details regarding the novelty of this work instead of the engineering efforts as the inhomogeneous approaches have been already reported in the previous report.*

Response: We thank Reviewer 2 for this important comment. The previously reported inhomogeneous pixel array [*Sci. Robot.* **8**, eade4698 (2023)] is fundamentally different from the present work in both purpose and design. This previous work aimed to capture high-resolution images in the center of the sensor surface by concentrating more pixels therein, which has nothing to do with offering anti-image distortion. In contrast, our strategy involves arraying pixels in a warped configuration in the same manner as images are warped on a curved sensor surface, enabling the corrective reversal of distorting optics. In this configuration, the pixel density is sparser in the center of the sensor surface. Also note that the pixel distribution in the present work follows a mathematical formulation described in the manuscript, whereas the previous work employs no mathematical formulation. We have clarified this in the main text as follows:

[Main text] "Before concluding this section, we note that while Ref. 33 also reports inhomogeneous distribution of pixels, this previous work is fundamentally different from the present work in

both purpose and design. Specifically, the previous work concentrated pixels more densely at the center of the sensor surface to achieve a higher resolution therein, has thus nothing to do with correcting image distortion, and employs no specific mathematical formulation for the inhomogeneous arraying of pixels."

Furthermore, in the caption of the new Supplementary Table 2 we provide in this revision to compare the specifications of our bioinspired camera vs. other bioinspired cameras (please see **Response to Reviewer 2, Comment #3** below), we also reiterate how the inhomogeneous pixel arraying in Ref. 33 (which is Ref. S8 in the Supplementary Information) differs from that in the present work.

Comment #3: *The authors should compare this system with the previously reported hardware for anti-distortion regarding power consumption, chip area, speed, anti-distortion robustness under different application scenarios, etc.*

Response: Following this advice, we now provide new Supplementary Table 2 as below, comparing the specifications of our and other bioinspired cameras. As seen in the table—and to the best of our knowledge––, there is no reported bioinspired camera with the anti-image distortion capability. On the other hand, if Reviewer 2 meant to compare our camera with the standard image processing pipeline with de-warping calculation in the digital back-end (Supplementary Fig. 2a), our camera is certainly free of power consumption and processing time associated with the de-warping calculation, which we now more clearly describe in Supplementary Fig. 2 with the revision of its figure and caption (please see **Response to Reviewer 2, Comment #4**). The quantification of the power consumption and processing time can vary greatly depending on the image size and the silicon technology node. The chip area associated with the de-warping calculation in the traditional image processing pipeline is even more difficult to pinpoint, as the same back-end digital processor would perform many functions, including the de-warping calculation.

[Supplementary Information]

Specification of bioinspired cameras							
**Bioinspired camera**	**Shape of image sensor**	**Number of pixels**	**Interconnect design**	**Optical system**	**Pixel distribution**	**Distortion correction**	**Ref**
Artificial human eye	Spherical	256	Pop-up	Single lens (Plano-convex)	Homogeneous	N/A	[S1]
Shape-tunable electronic eye	Spherical	256	Serpentine	Single lens (Plano-convex)	Homogeneous	N/A	[S2]
Artificial aquatic vision	Spherical	351	Serpentine	Single lens (Spherical)	Homogeneous	N/A	[S3]
Neuromorphic artificial eye	Spherical	31	Mesh	Single lens (Plano-convex)	Homogeneous	N/A	[S4]
Electrochemical biomimetic eye	Spherical	100	N/A	Single lens (N/A)	Homogeneous	N/A	[S5]
Hemispherical electronic eye	Spherical	676	Kirigami	Single lens (Plano-convex)	Homogeneous	N/A	[S6]
Shape-adaptive imager	Spherical	1024	Kirigami	Single lens (Plano-convex)	Homogeneous	N/A	[S7]
Artificial cuttlefish eye	Cylindrical	380	Straight line	Single lens (Spherical)	Inhomogeneous	N/A	[S8]
Anti-distortion bioinspired camera	Cylindrical	4096	Straight line	Single lens (Spherical)	Inhomogeneous	Configurational in-sensor computing	This work

Supplementary Table 2 | Comparison of the specifications of our bioinspired camera vs. other bioinspired cameras. Our camera not only has the highest number of pixels but also uniquely features the anti-image distortion capability. Ref. S8 also reports inhomogeneous distribution of

pixels, but this is to concentrate pixels more densely at the center of the sensor surface to achieve higher resolution therein, not to correct image distortion.

[Supplementary References]

S1. Ko, H. C. *et al.* A hemispherical electronic eye camera based on compressible silicon optoelectronics. *Nature* **454**, 748–753 (2008).
S2. Jung, I. *et al.* Dynamically tunable hemispherical electronic eye camera system with adjustable zoom capability. *Proc. Natl. Acad. Sci. U.S.A.* **108**, 1788–1793 (2011).
S3. Kim, M., *et al.* An aquatic-vision-inspired camera based on a monocentric lens and a silicon nanorod photodiode array. *Nat. Electron.* **3**, 546-553 (2020).
S4. Choi, C. *et al.* Curved neuromorphic image sensor array using MoS_2-organic heterostructure inspired by human visual recognition system. *Nat. Commun.* **11**, 5934 (2020).
S5. Gu, L. *et al.* A biomimetic eye with a hemispherical perovskite nanowire array retina. *Nature* **581**, 278–282 (2020).
S6. Zhang, K., *et al.* Origami silicon optoelectronics for hemispherical electronic eye systems. *Nat. Commun.* **8**, 1782 (2017).
S7. Rao, Z. *et al.* Curvy, shape-adaptive imagers based on printed optoelectronic pixels with a kirigami design. *Nat. Electron.* **4**, 513-521 (2021).
S8. M, Kim. *et al.* Cuttlefish eye–inspired artificial vision for high-quality imaging under uneven illumination conditions. *Sci. Robot.* **8**, eade4698 (2023).

Comment #4: *The comparison schematics of the conventional hardware strategy or architecture for anti-distortion and the proposed method should be provided for better understanding.*

Response: Following this advice, we have revised Supplementary Fig. 2 to more clearly compare the work-flow difference between the traditional and our image processing scheme.

[Supplementary Information]

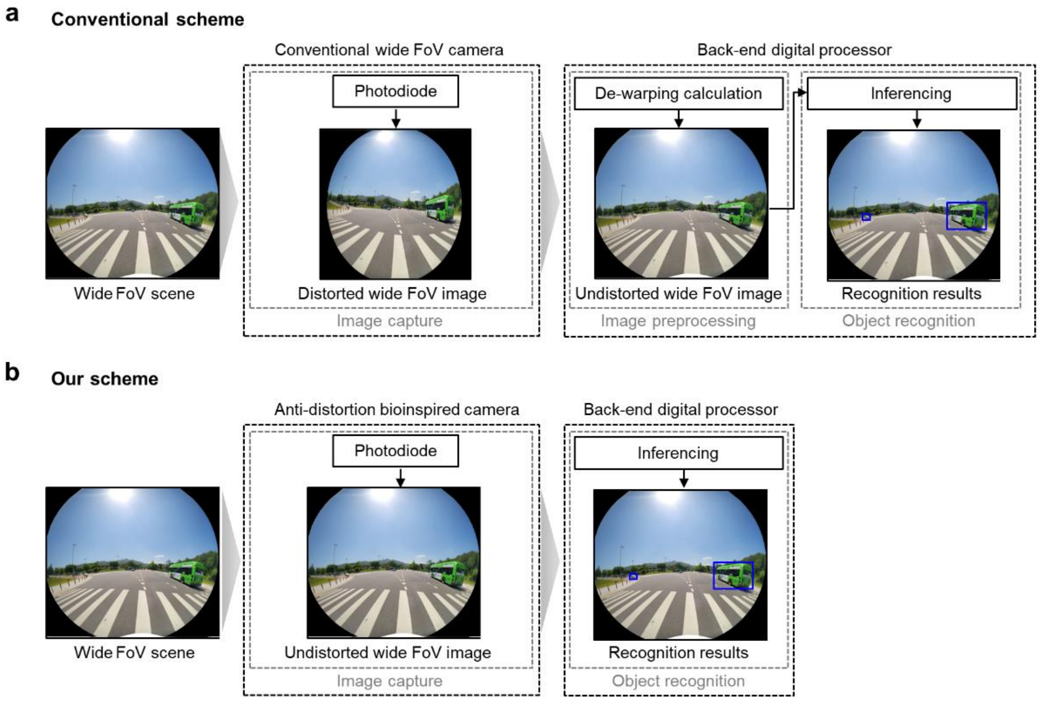

Supplementary Fig. 2 | Workflow difference for image distortion correction between conventional and our schemes. a, In the conventional scheme, a distorted image captured by the front-end image sensor is first sent to the back-end digital processor, wherein the distortion is first corrected by image de-warping software and then object recognition is performed. **b**, In our scheme, the front-end image sensor with the anti-image distortion capability captures an undistorted image. This would then be sent to the back-end digital processor, which would perform object recognition with no de-warping software. Thus our scheme is free of power consumption and processing time associated with the de-warping calculation. **Additional discussion.** In the context of the conventional image processing scheme (part **a**), object recognition might be able to be done with no de-warping calculation but with a more advanced—more computationally intensive—neural network that may recognize distorted images well, but this scheme would increase power consumption and processing time associated with the neural network inferencing, while removing those associated with the de-warping calculation. Which of the two schemes—no de-warping calculation with more computationally intensive neural network vs. de-warping calculation with less computationally burdensome neural network—would win depends on the details, but either scheme relying on the back-end digital processor for handling the image distortion would be worse in power consumption and processing time than our scheme (part **b**) where image recognition can be done with structurally-enabled anti-distortion image capture with less computationally burdensome neural network.

Comment #5: *The details of the neural network should be supplemented. Suppose a more robust neural network is used for accuracy comparison or the input images are very noisy or with a much larger size. Does the distortion correction really affect the network accuracies to a large degree?*

Response: First, following this advice, Methods of this revision now provides the CNN details, as follows:

[**Methods**] "Image recognition is performed using TensorFlow in Python 3.8. The CNN for the handwritten digit recognition (Fig. 5) starts with three convolutional layers. The first two convolutional layers includes 32 and 64 filters, each with 3 × 3 kernel size and ReLU activation function. Following each of these convolutional layers, max pooling is applied with 2 × 2 pool size. The third convolutional layer includes 64 filters, each with 3 × 3 kernel size and ReLU activation function. A flatten layer converts the resulting 3D feature maps into a 1D vector, which then undergoes two final layers sequentially: the first with 64 nodes and ReLU activation function, and the second with 10 nodes and softmax activation function. The CNN, trained over 20 epochs using 50,000 training images from the MNIST database, is used to recognize the handwritten digit images captured by our camera.

The CNN for the fashion image recognition (Supplementary Fig. 12) starts with three sets of convolutional layers. The first set includes two convolutional layers, each having 64 filters with 3 × 3 kernel size and ReLU activation function. The second set includes two convolutional layers, each having 128 filters with 3 × 3 kernel size and ReLU activation function. Following each set, max pooling with 2 × 2 pool size and batch normalization are applied. The third set is one convolution layer having 64 filters with 3 × 3 kernel size and ReLU activation function, and max pooling with 2 × 2 pool size is applied. The resulting 3D feature maps are converted by a flatten layer into a 1D vector, which is fed to two final layers sequentially: the first with 512 nodes and ReLU activation function, and the second with 10 nodes and softmax activation function. The CNN model trained over 20 epochs using 50,000 training images from the fashion-MNIST database is used to recognize the simulated fashion images."

Second, we agree that a more advanced—more computationally intensive—neural network might be able to recognize distorted images well with no correction, but this has an implication in power consumption and processing time. Concretely, object recognition with no de-warping calculation but with a more advanced neural network in the context of the conventional image processing scheme (Supplementary Fig. 2a) would increase power consumption and processing time associated with the neural net inferencing, while removing those associated with the de-warping calculation. Which of the two schemes—no de-warping calculation with a more advanced neural network vs. de-warping calculation with less computationally burdensome neural network—would win remains to be seen depending on the context, but either scheme relying on the back-end digital processor for handling the image distortion would be worse in power consumption and processing time than our scheme (Supplementary Fig. 2b) where image recognition can be done with structurally-driven anti-distortion image capture together with less computationally burdensome neural network. In this revision, we have included this consideration in the caption of Supplementary Fig. 2 as follows.

> **[Supplementary Information]** "**Supplementary Fig. 2**. .... **Additional discussion.** In the context of the conventional image processing scheme (part **a**), object recognition might be able to be done with no de-warping calculation but with a more advanced—more computationally intensive—neural network that may recognize distorted images well, but this scheme would increase power consumption and processing time associated with the neural network inferencing, while removing those associated with the de-warping calculation. Which of the two schemes—no de-warping calculation with more computationally intensive neural network vs. de-warping calculation with less computationally burdensome neural network—would win depends on the details, but either scheme relying on the back-end digital processor for handling the image distortion would be worse in power consumption and processing time than our scheme (part **b**) where image recognition can be done with structurally-enabled anti-distortion image capture with less computationally burdensome neural network."

Additional changes:

1. We have carefully gone through all the texts of the manuscript and edited them to improve English where relevant for better readability and clarity.
2. This revision has involved new experiments, and we have added the new contributors to these experiments to the author list.
3. Supplementary Fig. 8 and Fig. 3g have been re-simulated to reflect the setting of our camera (the original submission had an error in reflecting the setting of our camera).

REVIEWERS' COMMENTS

Reviewer #1 (Remarks to the Author):

The authors have addressed all my concerns. I recommend this paper to be accepted by Nature Communications now.

Reviewer #2 (Remarks to the Author):

The authors have addressed all the comments.I recommend the publication on Nature communications.

Point-by-Point Response to Reviewer 1

Comment #0: *"The authors have addressed all my concerns. I recommend this paper to be accepted by Nature Communications now."*

Response: We thank Reviewer #1 for the encouraging comment and valuable feedback on our manuscript.

Point-by-point Response to Reviewer 2

Comment #0: *"The authors have addressed all the comments. I recommend the publication on Nature communications."*

Response: We thank Reviewer #2 for the encouragement and the valuable comments.

Additional changes upon the editorial request:

1. We modified the heading of Conclusion into Discussion.
